# Interaction between the scaffold proteins CBP by IQGAP1 provides an interface between gene expression and cytoskeletal activity

**DOI:** 10.1038/s41598-020-62069-w

**Published:** 2020-04-01

**Authors:** Simone Kosol, Sara Contreras-Martos, Alessandro Piai, Mihaly Varadi, Tamas Lazar, Angela Bekesi, Pierre Lebrun, Isabella C. Felli, Roberta Pierattelli, Peter Tompa

**Affiliations:** 1 VIB Center for Structural Biology (CSB), Brussels, Belgium; 2 Structural Biology Brussels (SBB), Vrije Universiteit Brussel (VUB), Brussels, Belgium; 3 Magnetic Resonance Center, University of Florence, Florence, Italy; 4 Department of Chemistry “Ugo Schiff”, University of Florence, Florence, Italy; 5 Institute of Enzymology, Research Centre for Natural Sciences of the Hungarian Academy of Sciences, Budapest, Hungary

**Keywords:** Biophysics, Structural biology

## Abstract

Crosstalk between cellular pathways is often mediated through scaffold proteins that function as platforms for the assembly of signaling complexes. Based on yeast two-hybrid analysis, we report here the interaction between two complex scaffold proteins, CREB-binding protein (CBP) and the Ras GTPase-activating-like protein 1 (IQGAP1). Dissection of the interaction between the two proteins reveals that the central, thus far uncharacterized, region of IQGAP1 interacts with the HAT domain and the C-terminal intrinsically disordered region of CBP (termed ID5). Structural analysis of ID5 by solution NMR spectroscopy and SAXS reveals the presence of two regions with pronounced helical propensity. The ID5 region(s) involved in the interaction of nanomolar affinity were delineated by solution NMR titrations and pull-down assays. Moreover, we found that IQGAP1 acts as an inhibitor of the histone acetyltransferase (HAT) activity of CBP. In *in vitro* assays, the CBP-binding region of IQGAP1 positively and negatively regulates the function of HAT proteins of different families including CBP, KAT5 and PCAF. As many signaling pathways converge on CBP and IQGAP1, their interaction provides an interface between transcription regulation and the coordination of cytoskeleton. Disruption or alteration of the interaction between these scaffold proteins may lead to cancer development or metastatic processes, highlighting the importance of this interaction.

## Introduction

Many types of cancer arise from the misregulation of important cellular processes that are often governed by scaffold proteins. Molecular scaffolds are at the core of regulatory protein assemblies in which they frequently control post-translational modifications, stoichiometry and allosteric communication of effector molecules^1^. The crosstalk between cellular pathways, which may alter the direction of the flow of signaling information, is usually established through the direct interaction of scaffolding platforms^2,3^. Scaffold proteins are in the focus of an increasing number of studies, also motivated by their identification as potential therapeutic targets^4–6^.

**Figure 1:**
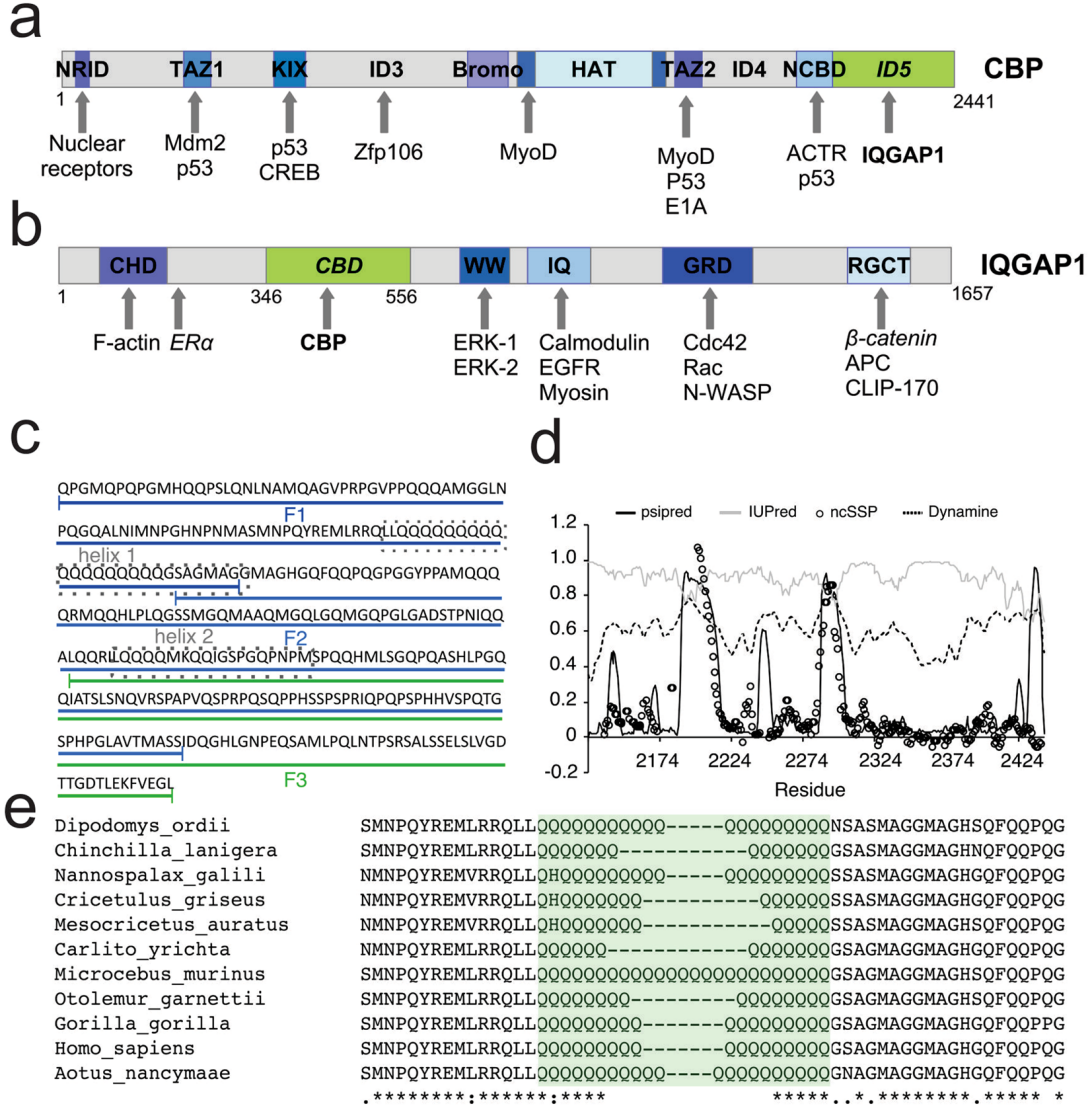
Domain architecture of CBP and IQGAP1. (**a**) domain architecture of CBP (UniProt Q92793) is shown with folded domains (colored regions) with a few selected interaction partners (out of 950 in Cytoscape, see text), linked by long IDR linkers (grey). Of these, ID3^11^ and ID4^44^ have been characterized in detail for structural disorder and functional interactions are marked. ID5 (aa2112–2442) covered in this study is shown in green. (**b**) Domain architecture of IQGAP1 (UniProt P46940) is also shown with characterized domains in color, along with some of their known partners (out of 627 in Cytoscape, see text). Proteins that have been described to also interact with CBP are highlighted in italics. The region identified in this study to be able to bind ID5 of CBP (termed CBP binding domain, CBD), is shown in green. CHD = calponin homology domain, CBD = CBP interacting region, IQ = IQ motif, WW = WW domain, GRD = GAP related domain, RGCT = RasGAP C-terminus, NRID = nuclear receptor interacting domain, HAT = histone acetyltransferase domain, NCBD = nuclear coactivator binding domain. (**c**) Sequence of ID5 fragments (ID5_F1 (aa2122–2223), ID5_F2 (aa2219–2395) and ID5_F3 (aa2291–2442), without the N-terminal His-tag and linker segment, used for signal assignment in NMR and interaction studies by pull-down and NMR, are shown. Two helical regions identified by NMR chemical-shift analysis (helix1 and helix2, cf. Fig. 4) are highlighted by dotted boxes. (**d**) Predicted and experimentally observed physical features of ID5: PsiPred^55^ and IUPred^53,54^ predict the tendency for structural disorder, DynaMine^56,57^ predicts local flexibility of amide bonds, whereas ncSSP^35^ shows the secondary structure propensity of the chain as derived from experimental chemical shifts. (**e**) Sequence alignments of the ID5 polyQ regions of homologs show that polyQ is present in all species but its length is not strictly conserved.

A classic example is the transcription coactivator CREB-binding protein (CBP), which coordinates and integrates diverse cellular processes such as differentiation, proliferation and apoptosis^7^. CBP consists of several domains that range from well-structured to less-defined molten-globule domains, mediating the interactions with a broad number of proteins (Fig. 1a). These well characterized functional domains are highly conserved, and are connected by sequentially more variable and structurally less characterized “linker” regions of different length that account for about half the total length of CBP. A region crucial for CBP function is its HAT domain, which can acetylate histones and a broad range of other substrate proteins^8^. Acetylation is a vital regulatory mechanism that, together with molecular scaffolding, allows CBP to precisely tune the expression of target genes via modulating protein-protein and protein-DNA interactions, DNA accessibility or subcellular localization^8^ in specific cellular functions, such as muscle differentiation or neurogenesis^9,10^.

Whereas folded domains of CBP have been implicated in functional interactions with other proteins, recently, we have shown that a long intrinsically disordered region (IDR) of CBP located between its KIX and bromodomains (termed ID3, cf. Fig. 1a) mediates protein-protein interaction with ZFP106, targeting the HAT activity of CBP on this newly identified partner^11^. Targeted and specific acetylation of ZFP106 has been implicated in mediating the cross-talk between transcription regulation and developmental and differentiation pathways.

Here, we set out to structurally and functionally characterize the long C-terminus of CBP (termed ID5, cf. Fig. 1a). We show by NMR and SAXS that it is an IDR with high propensity for local structure formation. In addition, by yeast two-hybrid (Y2H) screening of human placenta and fetal-brain libraries, we identified several proteins that interact with ID5, among them the cytoskeletal scaffold protein IQGAP1 (Fig. 1b). We further demonstrate that IQGAP1 regulates the HAT activity of CBP *in vitro*. IQGAP1 is a large multi-domain protein involved in cytoskeleton regulation and cell signaling, coordinating cytoskeletal functions as an enhancer of the Wnt pathway that controls cell proliferation, migration, differentiation and polarity^12^. For example, IQGAP1 binds and contributes to the normal transcriptional function of the estrogen receptor α (ER α)^13^, which has also been identified as an interaction partner of CBP. IQGAP1 is also involved in scaffold-scaffold protein interactions facilitating crosstalk between cellular signaling cascades^14^, as is also underscored by our study. The close relationship of the two proteins, CBP and IQGAP1, is also highlighted by them sharing many interacting partners (cf. Results and Suppl. Fig. S1). Of further significant biological relevance, IQGAP1 does not regulate acetylation by targeting the substrate (as we observed previously with ZFP106^11^), but directly inhibits the HAT activity of CBP. Furthermore, it has a more complex and varied role in protein acetylation regulation; while it also inhibits the p300/CREB binding protein (CBP)-associated factor (PCAF) HAT-domain, it enhances the HAT activity of KAT5.

These observations suggest that the interaction of CBP and IQGAP1 provides a new link between important signaling pathways impinging on CBP and the coordination of cytoskeleton activity. As the malfunctioning of both CBP^15,16^ and IQGAP1^17^ is involved in cancer, it is tempting to speculate that misregulation of their interaction and proper signaling/scaffolding function may also be involved in oncogenesis, presenting this interaction as a potential novel therapeutic target. A similar relation was previously suggested for the CBP/b-catenin interaction^18^.

## Results

### IQGAP1 interacts with CBP

First, we investigated if the long C-terminal region of CBP (aa2112–2442, termed ID5, cf. Fig. 1a) mediates interaction with other protein(s). The region, similarly to ID3^11^, is predicted by IUPred to be intrinsically disordered and carry some potential binding regions (cf. Fig. 1d). To this end, we carried out yeast two-hybrid (Y2H) experiments in which we screened cDNA libraries derived from human placenta and fetal-brain tissue with ID5 as a bait. Because CBP mediates the important function of CREB in early brain development^19^, we thought to identify relevant interactions of CBP by comparing hits in these two libraries. That is, placenta expresses a broad set of proteins representing early, non-differentiated state of cells, whereas fetal brain represents proteins involved in neuronal differentiation. In accord, we thought positive hits might be representative of the basic function(s) of this large scaffold protein.

In the fetal brain library, we found 51 strong hits, whereas in placenta, we found 14, probably reflecting on the primary function of CBP in early neuronal differentiation (Suppl. Table S1). Of these 65 hits, we selected IQGAP1 (Fig. 1b) for further interaction studies for several reasons. Firstly, both are scaffolds that coordinate the communication between a potentially very large number of proteins, the interaction of which is a largely unexplored area of research. Although IQGAP1 and CBP have not been shown to interact (in the BioGrid database), each have a very large number of interaction partners (CBP: 810, IQGAP1: 524), of which 38 are common (cf. Suppl. Fig. S1); this suggests that they must be functionally linked. Second, five different IQGAP1 hits, all with overlapping regions, provides strong evidence that this interaction takes place in one specific region. Third, both proteins are involved in cancer, thus their interaction may present an interesting target site. Finally, aside from IQGAP1, we made the recombinant expression in *E. coli* of several further hits (Suppl. Table S1) and tested their interaction with CBP *in vitro*; of these, IQGAP1 binding was the most confirmatory.

All five mentioned overlapping IQGAP1 fragments interacting with ID5 (Suppl. Fig. S2, Table S1) contained region aa346–556 of IQGAP1, to which no function has been assigned yet. Accordingly, we will refer to this region as CBP binding domain (CBD) of IQGAP1 (cf. Fig. 1b). The interaction was confirmed *in vitro* by pull-down assays with immobilized biotinylated ID5 and IQGAP1-F, a His-tagged IQGAP1 construct (residues 286–592 of IQGAP1) encompassing the CBP binding domain (aa346–556), and vice versa (Suppl. Fig. S3a).

The interaction was quantitatively characterized by Bio-layer interferometry (BLI), in which we immobilized ID5 and titrated it with IQGAP1-F (aa346–556, encompassing CBD, cf. Fig. 1b). Fitting of titration curves yields a K_d_ value of 0.4 ± 0.3 *μ*M for ID5 and IQGAP1-F (Suppl. Fig. S4). The binding of IQGAP1-F to full-length CBP is of similar strength (although binding assumes a more complex mechanism, cf. Suppl. Fig. S4), whereas its binding to the core region of CBP (from bromo- to Taz2 domain, aa1095–1849, cf. Fig. 1a) alone was much weaker (in the mM range, which, due to the lack of saturation in BLI, cannot be exactly determined). These observations suggest that binding of IQGAP1 at the two binding regions in CBP, i.e., the core region and ID5, do not cooperate (cf. inhibition experiments). This is probably due to the structural disorder of ID5, which makes the two binding events be mechanistically (and thermodynamically) largely isolated from each other.

### Structural characterization of the interacting domains

**Figure 2:**
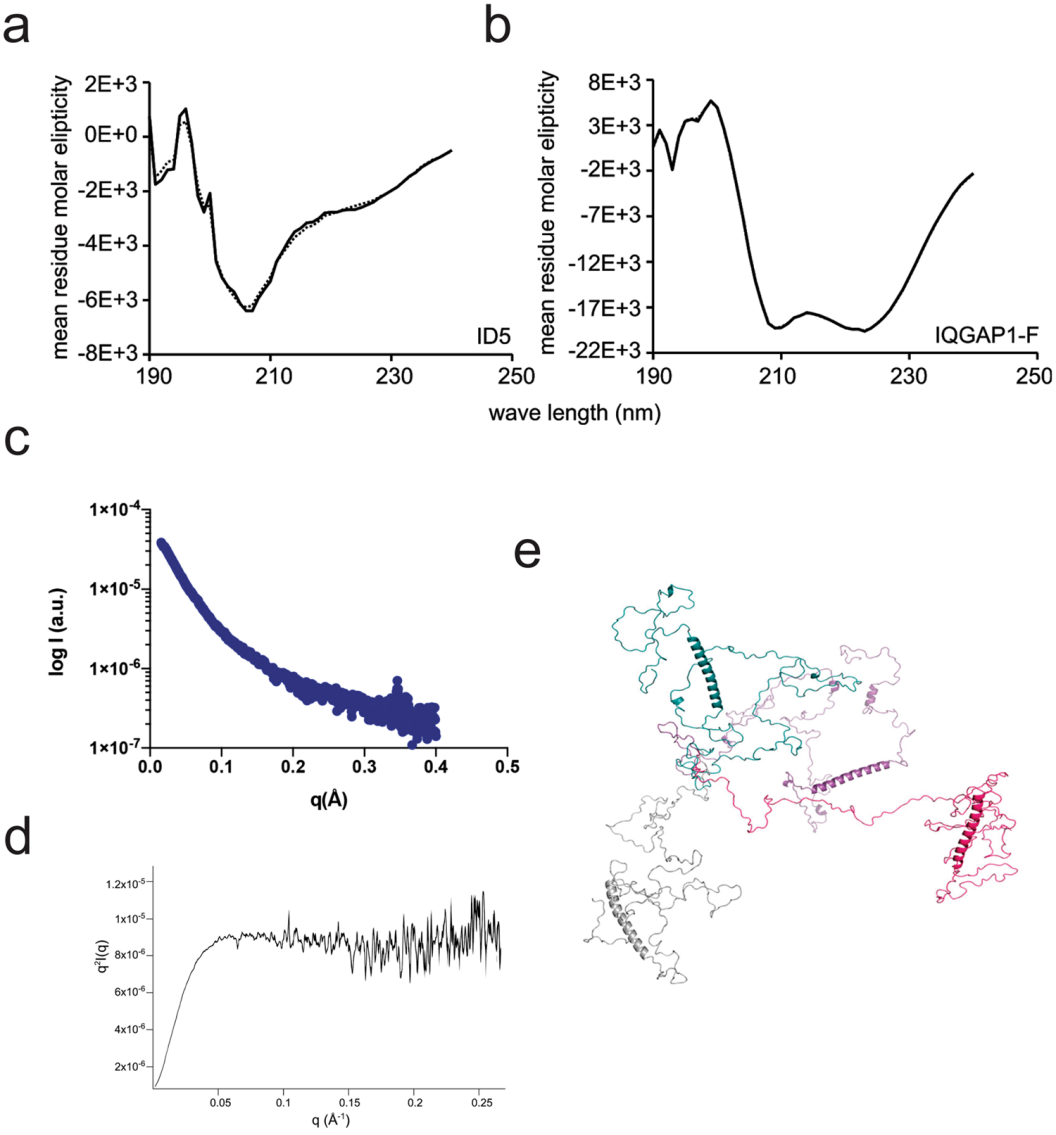
CD spectra of ID5 and IQGAP1-F and SAXS analysis of ID5. CD spectrum of ID5 (**a**) and IQGAP1-F (**b**). DichroWeb fits using CDSSTR data set 4 for ID5 and data 7 for IQGAP1-F domain architecture of CBP and IQGAP1 are represented as dashed lines. Mean residue molar ellipticity is given in deg*cm^2^/dmol. The scattering curve of SAXS analysis (**c**) and normalized Kratky plot (**d**) of the SAXS scattering of ID5. Fitting of data gives a radius of gyration (Rg) value of 50 Å, which is larger than that expected for a globular protein (21.7 Å) but smaller than of a fully disordered protein (76.5 Å) of the same M_w_. (**e**) A few representative conformations of the ensemble calculated from NMR and SAXS data show a dominant helical region (helix1), also apparent from other observation (cf. Figs. 1 and 4).

As outlined, most known interactions of CBP are mediated by its folded domains. Protein-protein interactions, however, can also be mediated by IDRs, most often by binding to folded partners in an induced folding process^20,21^. Less frequently, but not without a precedence, IDRs can also bind to each other in a process of mutual induced folding^22^, or even in a fuzzy interaction, when they remain disordered after binding^23^. This is what we have observed for the interaction of ID3 of CBP and ZFP106^11^. Along these lines, we set out to investigate the structural properties of the two identified interacting regions. Whereas ID5 is predicted to be intrinsically disordered (cf. Fig. 1d), IQGAP1 CBD appears ordered by several criteria. As shown (Suppl. Fig. S5) CBD falls into a region of IQGAP1 that has a significant predicted coiled-coil propensity, which is confirmed by circular dichroism (CD) spectroscopy. Analysis of the CD spectra of the two proteins (CBP: Fig. 2a, IQGAP1-F: Fig. 2b) by DichroWeb^24^ suggests around 30% helical content for ID5 and around 50% for IQGAP1-F. These data also agree well with secondary-structure predictions using PsiPred, DynaMine and IUPred (Fig. 1d).

We further characterized the structural properties of ID5 by solution NMR spectroscopy and small-angle X-ray scattering (SAXS). In parallel, we attempted to crystallize IQGAP1-F but could not obtain diffracting crystals. The SAXS experiments of ID5 (Fig. 2c,d) show a primarily disordered structure (by the shape of the Kratky curve, Fig. 2d), and, in accord, fitting of the data yielded a radius of gyration (Rg) value (50 Å) that is significantly larger than that expected for a globular protein (21.7 Å) of this Mw (355 amino acids, 38.29 kDa^25^). Its Rg, however, is smaller than that expected for a fully disordered random-coil (RC) chain (76.5 Å, cf.^26^), corroborating the finding by CD of the presence of a significant amount of secondary-structural elements in the protein (Fig. 2a). The importance of this observation derives from the concept that interactions of IDPs are often mediated by short motifs^27^, which may have a strong structural preference in isolation for the structure they assume in the bound state^28,29^.

To obtain residue-level secondary structure propensities, we turned to NMR spectroscopy. However, chemical-shift assignment of isotopically labelled [^13^C, ^15^N] full-length ID5 proved to be highly difficult due to severe signal overlap resulting from the presence of a polyQ region of 18 consecutive Gln residues and 75 Gln residues in the full construct (see Fig. 1c for the sequence). Further, the polyQ region promoted aggregation of ID5 samples at higher protein concentration and limited the sample concentration to 300 µM.

**Figure 3:**
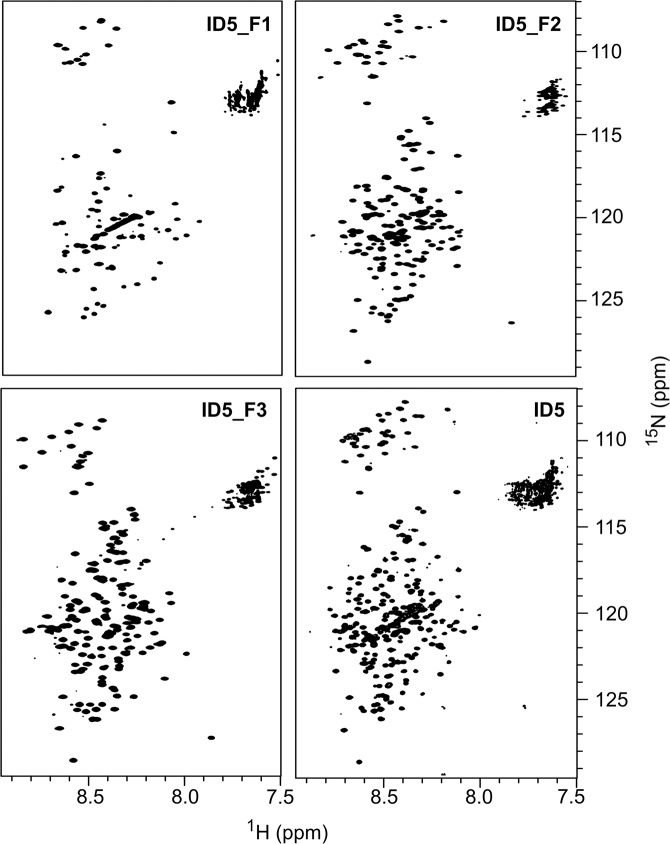
TROSY spectra of ID5 and its fragments. ^1^H-^15^N TROSY spectra of ID5 fragments ID5_F1 (aa2122–2223), ID5_F2 (aa2219–2395), and ID5_F3 (aa2291–2442), and of full-length ID5.

To overcome this limitation, we applied a divide-and-conquer approach by subdividing ID5 into three shorter overlapping constructs (ID5_F1, aa2122–2223, ID5_F2, aa2219–2395, and ID5_F3, aa2291–2442, cf. Fig. 1c), a strategy that has been used in the past to assign long and heterogeneous IDPs^30–32^. Resonances of the three fragments (Fig. 3) were assigned independently and aided in the assignment of the spectrum of full-length ID5 (Table S2). By splitting up the domain to shorter segments, the complexity of the system was reduced and only the fragment containing the polyQ (ID5_F1) suffered from aggregation at higher concentrations. Therefore, it was possible to assign 72% of the residues of ID5 (prolines constitute 13% of the sequence) using a set of 3D experiments tailored for IDPs^33,34^ (see Suppl. Tables S3–S6 for the exact experiments and experimental conditions). The lowest coverage of the sequence was achieved for the ID5_F1 fragment due to severe signal overlap, repetitive elements and very low signal intensities in some regions, thus the majority of missing assignments in ID5 fall into the first 100 amino acids. Comparison of C^𝛼^ and C^*β*^ chemical shifts between full-length ID5 and fragments show good agreement with only minor discrepancies (Suppl. Fig. S6). These discrepancies can be explained by the use of different NMR buffers that were required due to differing isoelectric points of the fragments. However, it cannot be ruled out that dividing ID5 may have some effect on long-range intramolecular interactions within the protein.

**Figure 4:**
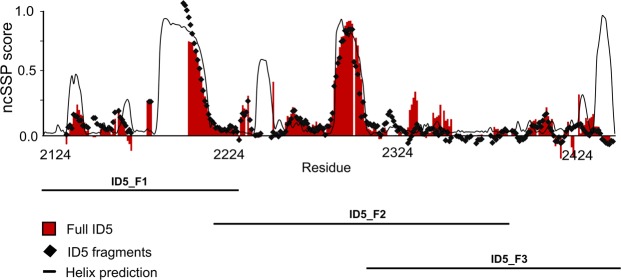
Neighbor-corrected secondary structure values of ID5 and its fragments. NcSSP values calculated using chemical shifts of the full-length ID5 (red bars) and fragments (black diamonds), compared with predictions by PsiPred for 𝛼-helices (black line).

Neighbor-corrected secondary-structure propensity scores (ncSSP)^35^ were calculated from the obtained chemical shifts of full-length ID5 and the three fragments (Fig. 4). Overall, the ncSSP scores of full-length ID5 agree well with the ncSSP scores calculated from the fragments indicating that the secondary-structural elements identified are reliable. ncSSP values suggest the presence of two α-helical regions in ID5, one at the C-terminus of ID5-F1 (helix1, aa2189–2211), and another in the middle of ID5_F2 (helix2, aa2287–2297). The chemical shifts place approx. 25% of the assigned residues in helical regions which, in good agreement with CD measurements (Fig. 2) and secondary-structure and dynamics predictions (Fig. 1d). As suggested, this observation could be of relevance for the interaction of ID5 with IQGAP1, pointing to potential preformed binding motifs within ID5^28,29^. The C-terminus of ID5 (residues 2428–2442), on the other hand, shows no helical propensity by NMR, in contrast to predictions (Fig. 4).

The presence of secondary-structural elements is further corroborated by relaxation parameters ^15^N R_1_ and R_2_ rates, and HetNOE values (Suppl. Fig. S7). The elevated R_2_ rates and HetNOE clearly show a reduced mobility in the two major helical regions, which is suggestive of potential binding sites in these regions. The overall picture that emerges from the NMR characterization of ID5 is that of a highly heterogeneous protein region (heterogeneity of peak intensities, salt dependence of chemical shifts, small changes identified when studying smaller constructs, etc.) with two well defined helical elements and a complex interplay of very weak and transient intramolecular interactions, not further characterized here.

### Identification of binding regions in ID5

Due to the potential preformed binding elements within ID5_F1, and ID5_F2, we carried out some bioinformatics analysis to ascertain if these regions have additional specific features indicative of their potential involvement in protein-protein interactions. To this end, we ran two dedicated predictors (MoRFpred^36^ and MoRFCHiBi^37^) on the sequences, and found that the helices show a significant potential to mediate protein-protein interaction(s) (Suppl. Fig. S8). This is most interesting in the case of helix1, which incorporates a long polyQ region with a preferred helical conformation. This motif is present in all other species studied (cf. Fig. 1e), but its length varies, as very often observed with homopolymeric repeats in proteins. It is of note that a particular class of transactivator domains of transcription factors is Gln-rich in nature^38,39^, and mediate protein-protein interactions, thus our observation may shed light on a general functional feature of such regions.

**Figure 5:**
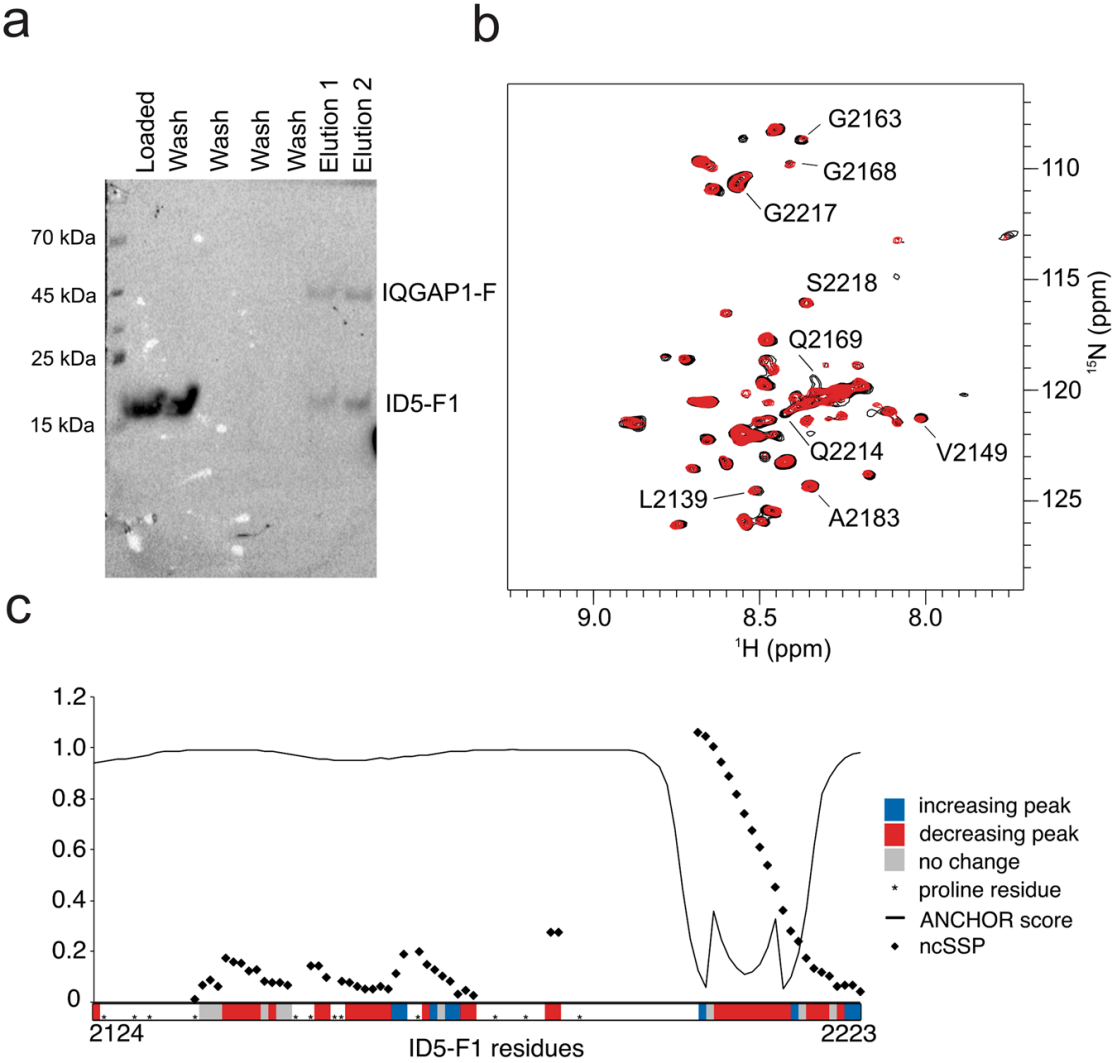
Interaction of ID5 and IQGAP1. (**a**) Anti-His Western blot of pull-down assay of biotinylated IQGAP1-F immobilized on Streptavidin beads and ID5_F1 (see also figure S3c). (**b**) Comparison of TROSY spectra of ID5_F1 alone (black) and ID5_F1 in the presence of equimolar IQGAP1-F (red). (**c**) Changes in D5_F1 peak volumes upon addition of IQGAP1-F. Residues with increasing peak volumes are labelled in blue, decreasing peak volumes in red and residues showing no significant changes in peak volumes are labelled in grey. The positions of prolines are indicated with asterisks (*). Experimental ncSSP scores (diamond symbols) and ANCHOR binding site predictions (black line) are plotted above.

As suggested by Y2H and pull-down experiments above, ID5 can specifically interact with the CBD region of IQGAP1. To identify and characterize the exact region(s) involved, and to address the potential importance of observed helix1 in ID5_F1, we conducted pull-down assays with three ID5 fragments (we used a shorter version of ID5-F2 to have only short sequence overlaps, Fig. S3c). Of the three fragments, only ID5_F1 binds sufficiently strong to elute together with immobilized biotinylated IQGAP1-F (Fig. 5a), suggesting that the binding region(s) localize in the region aa 2122–2223: this would be in line with the tendency of preformed helix1 to mediate protein-protein interaction (cf. Fig. 4 and Suppl. Fig. S8).

Therefore, we titrated ^15^N-labeled ID5_F1 with IQGAP1-F to identify residues involved in the interaction by NMR (for experimental conditions, cf. Suppl. Table S7) and observe potential structural changes as a consequence of the interaction. We observed several disappearing resonances upon the addition of IQGAP1-F, indicating intermediate-to-slow exchange on the NMR time-scale (Fig. 5b). However, no new peaks appeared, probably due to the size of the complex formed. It was not possible to fit the decay of peak intensity as a function of IQGAP1-F concentration, as the signals behaved relatively irregularly with some resonances gaining intensity while others disappearing (Fig. 5c and Suppl. Fig. S9) indicating a more complex binding mechanism. Our experience with NMR titration of a closely related IDP (the ID3 region of CBP^11^) is that changes in peak intensities, rather than chemical shift perturbations, are typical of intermediate-to-slow exchange which, in general, indicate affinities in the low micromolar to nanomolar range.

Here, the changes are observed to affect several regions over the full length of ID5_F1 including the helical region falling between residues 80–100 of ID5_F1 (aa2203–2223 of ID5, including part of the long polyQ element (aa2199–2216)). While the structural ensemble calculated from NMR and SAXS data (Fig. 2e) is dominated by the helical region (helix1), which is also apparent in other calculations (e.g. Figs. 1, 4 and Suppl. Fig. S8), the titrations suggests that elements with minor helical propensities are also involved in the binding (see Fig. 5).

### Functional role of the interaction between IQGAP1 and CBP

**Figure 6:**
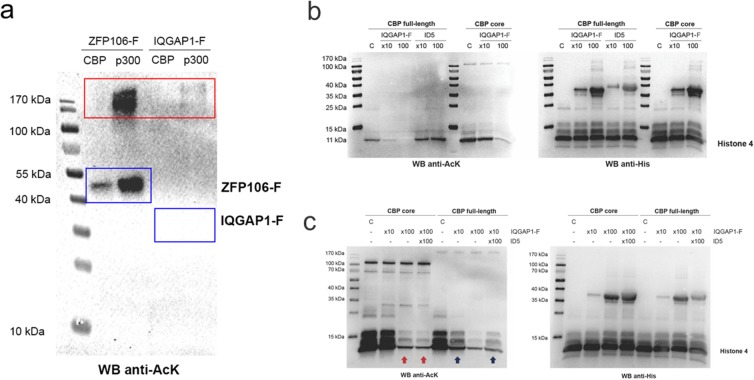
IQGAP1-F affects the acetylation activity of CBP. To evaluate if the HAT inhibitory function of IQGAP1-F was modulated by binding to ID5, acetylation reactions of H4 were performed in presence and absence of IQGAP1-F and ID5. (**a**) Western blot with anti-acetyl-lysine (anti-AcK) antibody of HAT assay results with the CBP substrate ZFP106-f^11^ and IQGAP1-F. Blue boxes indicate where ZFP106-f and IQGAP1-F are expected to appear, whereas the red box indicates CBP/p300. (**b**) Western blots of acetylation reactions with Histone 4 as substrate, upon the addition of either IQGAP1-F or ID5 at various molar ratios (left panel with anti-AcK antibody, right panel with anti-His-tag antibody. C indicates control reaction, without IQGAP1-F). (**c**) Western blots of acetylation reactions with Histone 4 as substrate with IQGAP1-F and ID5 added in the same mixtures. No differences in the acetylation level of H4 by the CBP full-length were observed when adding 10-fold excess of IQGAP1 or 10-fold excess of IQGAP1 plus 100-fold excess of ID5 (blue arrows). The same observations were made in case of the CBP core domain (red arrow). Please note that higher amount of IQGAP1-F was required for a significant inhibition of the core domain.

CBP/p300 is a transcriptional signal integrator that mainly functions as a platform for the assembly of multiprotein complexes, which then target the HAT activity of CBP/p300 on histones and other types of proteins^7^. In a previous work, we reported that its IDR ID3 domain targets CBP HAT activity on its binding partner ZFP106. In earlier studies, mouse IQGAP1 was found to be acetylated^40^, therefore, we asked if the region that binds ID5, IQGAP1-F, could also be a substrate of CBP. In acetylation assays with active, full-length CBP (and p300), however, no acetylation of IQGAP1-F was observed (Fig. 6a), as also confirmed by further experiments and assay combinations (e.g. Fig. 6b,c). Unexpectedly, auto-acetylation of CBP and p300, which is generally apparent under such assay conditions, is significantly suppressed in the presence of IQGAP1-F (Fig. 6a), which may indicate that IQGAP1 regulates the HAT activity of CBP.

To further investigate this effect, we performed a range of acetylation reactions using full-length CBP and its core domain on the canonical CBP substrate, histone 4 (H4)^41^. Acetylation reactions were carried out in the absence, or presence, of increasing amounts of IQGAP1-F (10× and 100×, relative to the CBP concentration). In the case of full-length CBP, H4 acetylation was already significantly reduced by a 10-fold excess of IQGAP1-F, and completely abolished when it was added in a 100-fold molar excess (Fig. 6b). A notable inhibition of the HAT activity of the core domain of CBP (from bromo- to Taz2 domain, aa1095–1849, cf. Fig. 1a) was only achieved with a 100-fold excess of IQGAP1-F. Note, that in our experiments, the acetylation activity of the core domain is generally higher compared to full-length CBP.

### Mechanism of CBP inhibition by IQGAP1-F

Since IQGAP1-F can bind to ID5 of CBP, we wondered if the two regions (ID5 and the HAT domain) cooperate in IQGAP1-F binding, resulting in the targeted and specific inhibition of the catalytic center. To probe if this is the case, we first measured if ID5 (alone) has a direct effect on the HAT activity of full-length CBP. However, ID5 in excess did not cause any observable difference in H4 acetylation (Fig. 6b).

To assess the potential of ID5 in targeting IQGAP1-F-mediated inhibition, we next added an excess of ID5 into an acetylation reaction inhibited with IQGAP1-F. We reasoned if ID5 was directly involved in mediating acetylation inhibition by IQGAP1-F, competition by added ID5 would reduce the inhibitory effect and at least partially restore acetylation activity. However, when recombinant ID5 was added in 10x excess over IQGAP1-F to the H4 acetylation reaction with full-length CBP, no rescue effect was observed and the acetylation level of H4 was similar to that observed in the absence of ID5 (Fig. 6c). These results suggest that the interaction between ID5 and IQGAP1-F is not the basis of the inhibition of the HAT activity, i.e., IQGAP1-F probably has additional binding site(s) that mediate its direct interaction with the HAT domain.

### IQGAP1 modulates the activity of other HAT-domain containing proteins

To further understand the mechanism of HAT inhibition by IQGAP1-F, we also conducted acetylation reactions with other HAT enzymes. HATs are classified into three main families^42^ by structural and functional criteria, in this classification, CBP and p300 constitute their own family called KAT3 (see Suppl. Fig. S10).

**Figure 7:**
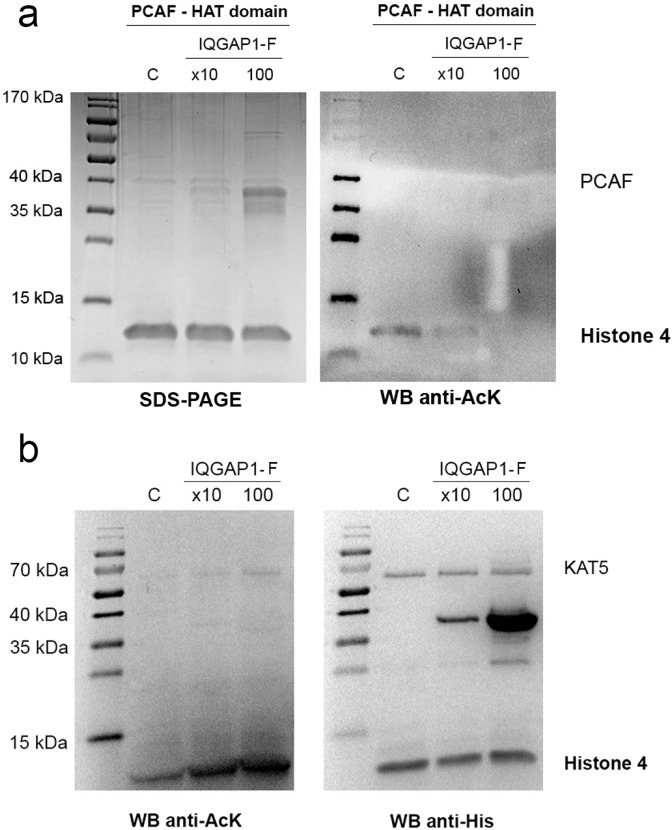
IQGAP1-F inhibits PCAF but activates KAT5 HAT activity. The effect of IQGAP1-F on the HAT activity of two homologues of CBP, PCAF (**a**) and KAT5 (**b**) was tested in Western blots developed by anti-AcK (left panel) and anti-His-tag (right panel) antibodies. Acetylation reactions were run in the absence (marked C) and presence of increasing amounts (x10, 100; relative to CBP concentration) of IQGAP1-F (40 kDa). A significant decrease in H4 acetylation by PCAF was observed in presence of 10- or 100-fold excess of IQGAP1-F, however, the activity of KAT5 enzyme was enhanced in presence of IQGAP1-F, in a concentration-dependent manner.

To this end, the commercially available HAT domain of p300/CBP-associated factor PCAF or KAT2B (belonging to the GNAT family) was used to acetylate H4 in the absence and presence of IQGAP1-F. Interestingly, IQGAP1-F was able to strongly inhibit the PCAF HAT domain (Fig. 7a), which suggest a general HAT-regulatory effect of IQGAP1.

To probe further into this IQGAP1 function, we also tested its effect on another HAT protein from a different family, the 60 kDa Tat-interacting protein (KAT5 or so-called KAT5; from the MYST family). Unexpectedly, a concentration-dependent enhancement of the KAT5 HAT activity was observed in the presence of IQGAP1-F (Fig. 7b). These results of acetylation assays with different HAT domains suggest a regulatory role for IQGAP1 in protein acetylation.

## Discussion

CBP/p300 are large signaling proteins of about 2400 residues in length, containing several conserved functional domains (for example, TAZ1, KIX and HAT domain) connected by long, much-less characterized linker regions (Fig. 1). Whereas structurally characterized domains have been shown to mediate interactions with more than 400 partners in signal-integration and transcription functions^43^, much less is known about the structural status and potential functions of the long linker regions, although they make up about half the total length of the protein. In a series of studies, we have set out to analyzing the structure and potential interaction function of three such regions.

Predicted structural disorder and functional interaction with ZFP106 (and possibly other proteins) have been experimentally demonstrated for the region termed ID3 (aa674–1080)^11^, whereas structural disorder and the presence of pre-structured potential binding elements was shown for another region termed ID4 (aa1851–2057)^44^. Other such regions of CBP/p300 have not been studied in detail yet, although IDPs/IDRs are known to often harbor short motifs mediating functional protein-protein interactions^21,27^. For example, long IDRs have been shown to engage in protein-protein interactions in the case of RNAse E^45^, BRCA1^46^, androgen receptor^47^ or UPF2^48^. Accordingly, we have set out to characterize the structural properties and possible functional interactions of another presumed IDR of CBP, ID5 (aa2112–2442), located at the C-terminus of the protein. Structural analysis of ID5 by a combination of solution NMR and SAXS confirmed that it is an IDR, whereas detailed analysis of its chemical shifts and description of its structural ensemble suggests the presence of two regions (aa2189–2211 and aa2287–2297) with pronounced helical propensity. Interestingly, the first helix involves a polyQ region preceded by two leucine residues, a module that has recently been observed in the N-terminal disordered domain of a transcription factor androgen receptor^38^ - it will be interesting to see if this is the prototype of a general interaction motif. The importance of this observation derives from the general notion that pre-structured regions in the structural ensemble often delineate specific binding regions of the IDP^28,49^.

Next, we applied a Y2H screen to identify potential binding interactions mediated by ID5 and found several potential hits. Of these, we carried out a detailed analysis of the mode of its interaction with the Ras GTPase-activating-like protein 1 (IQGAP1), with which CBP1/p300 have 38 common interacting partners, and its potential functional consequences. The interaction with ID5 was confirmed by pull-down assays and BLI. By NMR-based titration, we found that the interaction is primarily mediated by one of the pre-structured alpha-helices of ID5 (aa2203–2223 within CBP, corresponding to the C-terminus of the construct ID5_F1). In addition, IQGAP1 is not only an interaction partner of ID5, it is also an inhibitor of the HAT activity of CBP. In addition, this region of IQGAP1 also regulates the function of HAT proteins that belong to other families, inhibiting PCAF but activating KAT5.

IQGAP1-F binds to ID5 and at least one other domain present in the CBP core region (bromodomain, PHD, RING, ZZ and/or TAZ2) which suggests that the regulation mechanism might be rather complex. This is in accord with IQGAP1-F inhibiting the HAT domain of a closely-related enzyme, PCAF. It is of note that huntingtin (Htt) protein, associated with the neurodegenerative disease Huntington’s disease, was observed to interact “*in vitro*” with the HAT domains of PCAF, CBP and p300 and to inhibit their HAT activity^50^. IQGAP1 function, however, seems to go beyond simple inhibition, as witnessed by the activation of the HAT domain of KAT5, a more distant member of the enzyme family. It seems possible that IQGAP1-F binding to different HAT domains causes conformational changes that either facilitate or block access of substrate(s) to the active sites of the enzymes. Indeed, studies involving the type 1-encoded TAT protein (Trans-Activator of Transcription), which was described to selectively inhibit substrate acetylation by CBP, KAT5 and GCN5, suggested that the inhibitory action of TAT depends on the structure of the substrate and on the TAT-induced conformational changes in the enzyme^51^.

Quantitative aspects of the effect of IQGAP1 on the HAT domain of CBP cast an interesting mechanistic picture. The binding and inhibition experiments suggest two independent IQGAP1 binding regions in CBP: (at least) one in ID5 and another one in the HAT region. In terms of thermodynamics, ID5 seems to dominate binding, as it is much stronger than that of the HAT region. Due to the possible cooperation between two binding regions of CBP connected by a disordered linker region, one would expect an even stronger binding when both are present (on full-length CBP). This is not the case, though: the difference between binding of full-length CBP and the ID5 region is small, suggesting that the two binding events are isolated from each other, i.e. they show very little – if any – cooperativity. In mechanistic terms, cooperativity of different binding sites results from the avidity effect, which is the result of the high effective local concentration, when two binding elements are physically connected^52^. In our case, however, the length and structural disorder of the region linking the two binding motifs (actually on both proteins) however, enables one binding motif to remain mobile, exploring a large space after binding through the other, which is well exemplified by fuzzy interactions, i.e. interactions occurring without induced folding^23^. Such an interaction limits the entropic penalty of the second binding only marginally, making the two events thermodynamically independent.

This picture is in line with the inhibition experiments. If we assume that the primary binding of IQGAP1 at ID5 and its inhibitory binding around the HAT domain cooperate, IQGAP1 should be a potent inhibitor of HAT activity of full-length CBP. In fact, for a significant inhibition of CBP, a large excess of the inhibitor (100×) is required with the HAT region of the enzyme, however, and even with the less active full-length CBP, a 10x excess of the inhibitor is not sufficient for the full inhibition of its acetyl-transferase activity. Titration of the IQGAP1-inhibited systems with ID5 provides further evidence for this scenario. If strong binding to ID5 was critical for inhibition, titration of ID5 in activity assays should have resulted in significantly reduced inhibition. This was not the case which indicates that binding of free ID5 region does essentially not interfere with binding at the HAT region – leading to the conclusion that the two binding events are mechanistically and thermodynamically isolated. It also cannot be excluded that ID5-IQGAP1 binding mediates some function, unrelated to the regulation of acetyl transferase activity. In all, the binding of IQGAP1 to CBP exemplifies a fuzzy interaction^23^, in which binding is not accompanied by (complete) folding of the partner(s).

In a broader biological context, these observations point to the complex interplay of the action of the two scaffold proteins, which may rely on a combination of inhibition of HAT activity and probably also localization effects by stronger binding through ID5. Taking also into consideration the primary function of IQGAP1 in cytoskeleton regulation^14^ and that of CBP in transcription^7^, and the effect of IQGAP1 on other HAT enzymes, our results imply the interplay of protein acetylation with transcription regulation and cytoskeleton organization.

## Materials and Methods

### Sequence analysis

Structural disorder was analyzed using the IUPred online tool^53,54^. IUPred in “long” running mode was used to predict the disorder level; other parameters were left as default. Additionally, PsiPred^55^ was used to predict secondary structure propensities of the disordered domain, and the presence of local flexibility was analyzed using DynaMine^56,57^. Potential binding motifs were predicted by ANCHOR^58^ and MoRFpred^36^.

### Cloning

cDNA of full-length human CBP (UniProt Q92793) was amplified by PCR from synthesized CBP cDNA (Origene - RC219036), cloned in pDONOR221 and transferred to pDEST10 using Gateway® Cloning Technology (Life Technologies). The gene fragment encoding the IDR ID5 (residues 2124–2442 of CBP) was sub-cloned into pET200D via TOPO cloning or into pGEX4T1 using the restriction sites *BamHI and XhoI*. The genes encoding shorter fragments of ID5 ID5_F2 (aa2219–2395), ID5_shortF2 (aa2219–3206), and ID5_F3 (aa2291–2442) were sub-cloned into pet200D via TOPO cloning. ID5_F1 (aa2122–2223) was cloned into pet16b using the restriction sites *BamHI and XhoI*. The cDNA for the core domain of CBP (residues 1095–1849) was sub-cloned into pDEST10 via TOPO cloning. The DNA of IQGAP1 (UniProt P46940) fragment_1 (residues 286–592, IQGAP1-F) DNA received from Hybrigenics was PCR-amplified and cloned into pet200D via TOPO cloning. The constructs were sequenced before transformation into the *E. coli* expression strain BL21star.

### Yeast two-hybrid screen

Yeast two-hybrid (Y2H) screening was performed by Hybrigenics Services S.A., Paris, France (http://www.hybrigenics-services.com). In brief, ID5 coding sequence of CBP (encompassing aa2109–2442, GenBank accession number gi: 119943103) was PCR-amplified and cloned into pB27 as a C-terminal fusion to LexA (N-LexA-ID5-C). The construct was sequenced before being used as a bait to screen random-primed human placenta- and fetal-brain cDNA libraries. In total, 180.5 million interactions were analyzed. After selection on a medium lacking leucine, tryptophan and histidine, 436 clones were finally processed. The prey fragments were amplified by PCR and sequenced for identification of the corresponding interacting proteins.

### Database search for CBP and IQGAP1 interaction partners

Interaction partners of CBP (Q92793) and IQGAP1 (P46940) were retrieved by database search with their UniProt accessions in Cytoscape 3.5 that has access to 34 interaction databases^59^. For the interaction partners of human CBP and IQGAP1, they were restricted to protein-protein interactions (PPI) and then cross-species interactions were filtered out by selecting only human (taxonomy ID: 9606) proteins.

### Expression and purification of full-length CBP and its core domain

Full-length CBP and the core domain of CBP was expressed in Sf9 insect cells^41^ and purified similarly by consecutive Nickel- (His-select, Sigma-Aldrich) and anti-FLAG- (Sigma-Aldrich) immunoaffinity chromatography followed by size-exclusion chromatography in the presence of protease inhibitors (2 μg/ml leupeptin, 1 μg/ml bestatin, 1 g/ml pepstatin A, 5 μg/ml E64, and 2 μM 4-(2-aminoethyl)benzenesulfonyl fluoride hydrochloride (AEBSF)). Chelating agents were avoided to keep the Zn-finger domains of CBP intact. The purified protein was flash frozen in liquid nitrogen and stored at −80 °C in storage buffer (50 mM Tris pH 7.5, 150 mM NaCl, 5% glycerol, 50 μM tris(2-carboxyethyl)phosphine (TCEP), and protease inhibitors: 1 μg/ml leupeptin, 0.5 μg/ml bestatin, 0.5 μg/ml pepstatin A, 1 μg/ml E64, and 0.1 μM PMSF.

### Expression and purification of other protein constructs

ID5, ID5_F1, ID5_F2, ID5_F3, and IQGAP1-F were generated as follows (for exact constructs, cf. Suppl. Table S8). Transformed *E. coli* BL21(DE3) (Invitrogen) cells were grown in Luria Broth (LB) medium at 37 °C until their optical density (OD) reached 0.6, when protein expression was induced with 1 mM IPTG. For [^15^N-^13^C] labelled protein samples, cells were grown to a high cell density and centrifuged (15 min, 3500 g); pelleted cells were resuspended in M9 medium and the culture was grown for 2.5 h at 37 °C before induction^60^. In all the cases, cell cultures were harvested after overnight expression at 25 °C for all samples. The pelleted cells were resuspended in lysis buffer (50 mM KH_2_PO_4_, 300 mM NaCl, 10 mM imidazole, 1 tablet of EDTA-free protease inhibitor cocktail (Roche), DNase, lysozyme, pH 8.0), sonicated and followed by heat-treatment (85 °C, 10 min, heat-treatment causes no structural changes, cf. Suppl. Fig. S11), in case of all ID5 samples^61^. Cell lysates were cleared by centrifugation at 13000 g for 45 min. Subsequent purifications were done by IMAC (HisTrap HP or GSTrap FF column; GE Healthcare) followed by size-exclusion chromatography (Superdex200 26/600 and Superdex75 26/600; GE Healthcare) in a buffer 50 mM KH_2_PO_4_, 150 mM NaCl, 1 mM DTT, pH 7.5, complemented by 2 tablets of EDTA-free protease inhibitor cocktail (Roche). Following buffer exchange into distilled water (dH_2_O), the sample was dried at room temperature (RT) in a vacuum concentrator (ID5) or lyophilized (IQGAP1-F) and stored at −20 °C or concentrated and stored at −80 °C.

### Small-angle X-ray scattering experiments

Small-angle X-ray scattering (SAXS) experiments were performed on the SWING beamline at the SOLEIL synchrotron (λ = 1.03 Å). The Aviex charge-coupled device detector was positioned at 1507.5 mm. A total volume of 60 µl of a ID5 sample (10 mg/ml) was injected into a size-exclusion column (SEC-3, 300 Ǻ Agilent), using an Agilent HPLC system, and eluted directly into the SAXS flow-through capillary cell at a flow rate of 0.2 ml min^−1^ and at 15 °C^62^. The size-exclusion buffer was 50 mM Tris, 150 mM NaCl_2,_ 5 mM TCEP, pH 6.5. SAXS data were collected continuously, with a frame duration of 1.0 s and a dead time of 0.5 s between frames. Selected frames corresponding to the main protein elution peak were averaged using FOXTROT^62^. A large number of frames were collected during the void volume of the elution and averaged to account for buffer scattering, which was subsequently subtracted from the signals during the protein elution. Data reduction to absolute units, frame averaging and subtraction were done using FOXTROT^62^. All subsequent data processing, analysis and modelling steps were carried out with the ATSAS suite^63^. The radius of gyration (*R*_G_) was derived by the Guinier approximation *I*(*q*) = *I(*0) exp(−*q*^2^*R*_G_^2^/3) for *q*R_G_ < 1.3 using PRIMUS QT^64^. GNOM was used to compute the pair-distance distribution functions, P(r)^65^. This approach also features the maximum dimension of the macromolecule, *D*_max_. Normalized Kratky plots (i.e. (*qR*_*G*_)^2^I(q)/I(0) as a function of qR_G_) were used to assess the conformational behavior of the polypeptide chain.

### NMR sample preparation

For NMR experiments, samples were prepared as follows. ID5: 0.3 mM ^13^C,^15^N labeled full-length ID5 (CBP residues 2124–2442), was dissolved in 50 mM Tris, 100 mM NaCl, 0.02% NaN3, pH 6.0 (measured at 283.0 K). ID5_F1: 0.6 mM of ^13^C,^15^N labeled protein (residues 2122–2223) dissolved in 50 mM Pi, 100 mM NaCl, pH 7.5 (measured at 278.0 K). ID5_F2: 0.5 mM ^13^C,^15^N labeled protein (residues 2219–2395) dissolved in 50 mM Tris, 100 mM NaCl, 0.02% NaN_3_, pH 6.0 (measured at 283.0 K). ID5_F3: 1.6 mM ^13^C,^15^N labeled protein (residues 2291–2442) dissolved in 200 ml 50 mM Tris, 100 mM NaCl, 0.02% NaN_3_, pH 6.0 (measured at 283.0 K).

### NMR data acquisition

2D and 3D BEST-TROSY (BT) NMR experiments^34,66^ were performed on samples ID5, ID5_F1 and ID5_F3 at 21.1 T on a Bruker Avance spectrometer operating at 898.57 MHz ^1^H, 225.95 MHz ^13^C and 91.05 MHz ^15^N frequencies, equipped with a cryogenically cooled probehead. PC9 and E-BURP2 (or time reversed E-BURP2) shapes of durations of 1800 and 1270 ms, respectively, were employed for ^1^H band-selective π/2 flip angle pulses^67^; REBURP shape of duration of 1250 ms was used for ^1^H band-selective π flip angle pulse^67^; BIP-750–50–20 pulse shapes of duration of 140 ms were used for broadband ^1^H inversion^68^. For ^13^C band-selective π/2 and π flip angle pulses G4 (or time reversed G4)^69^ and Q3^70^ shapes of durations of 274 and 190 ms, respectively, were used, except for the π pulses that should be band-selective on the C^α^ region (Q3, 660 ms). The ^13^C band selective pulses on C^α^ and C′ were applied at the center of each region, respectively. All gradients employed had a smoothed square shape.

3D BT-NMR experiments^34,66^ on sample ID5_F2 were acquired at 22.3 T on a Bruker Avance III spectrometer operating at 950.20 MHz ^1^H, 238.93 MHz ^13^C and 96.28 MHz ^15^N frequencies, equipped with a cryogenically cooled probehead. PC9 and E-BURP2 (or time reversed E-BURP2) shapes of durations of 1620 and 1200 ms, respectively, were employed for ^1^H band-selective π/2 flip angle pulses^67^; REBURP shape of duration of 1180 ms was used for ^1^H band-selective π flip angle pulse^67^; BIP-750–50–20 pulse shapes of duration of 200 ms were used for broadband ^1^H inversion^68^. For ^13^C band-selective π/2 and π flip angle pulses G4 (or time reversed G4)^69^ and Q3^70^ shapes of durations of 260 and 161 ms, respectively, were used, except for the π pulses that should be band-selective on the C^α^ region (Q3, 667 ms). The ^13^C band selective pulses on C^α^ and C′ were applied at the center of each region, respectively. All gradients employed had a smoothed square shape.

The NMR titration between the full-length ID5 and IQGAP1-F was performed at 21.1 T on a Bruker Avance spectrometer operating at 898.57 MHz 1 H, 225.95 MHz 13 C and 91.05 MHz 15 N frequencies, equipped with a cryogenically cooled probehead. A 2D BEST-TROSY experiment was acquired for each of the following ID5:IQGAP1 ratios: 1:0; 1:0.25; 1:0.5; 1:1; 1:1.75; 1:3.25. The concentration of ID5 was 0.1 mM. The NMR titration between ID5_F1 and IQGAP1-F was performed at 16.4 T on a Bruker Avance spectrometer with a cryogenically cooled probehead. 2D BEST-TROSY spectra were recorded for each titration point at ID5_F1:IQGAP1 ratios 1:0; 1:0.05; 1:0.1; 1:0.2; 1:0.5; 1:1. The concentration of ID5-F1 was 0.1 mM.

The most relevant experimental parameters used for the acquisition of all the NMR experiments are collected in the Supplementary Tables S3–S6. All the data sets were acquired using *Bruker TopSpin 1.3* or *3.1* software. The 3D experiments for sequence-specific assignment were performed using on-grid non-uniform sampling (NUS). The on-grid “Poisson disk” sampling scheme^71^ was chosen to generate the time schedules with the *RSPack* program. The distribution was relaxation-optimized, i.e. the density of points was decaying according to the Gaussian distribution *exp(−t*^2^*/σ*^2^), with *σ* = *0.5*.

### NMR data processing and analysis

Conventionally-sampled NMR data sets were processed with the software *Bruker TopSpin 1.3*. Instead, when NUS was employed, the NMR data were converted with *nmrPipe*^72^ and then processed using the Multidimensional Fourier Transform (MFT) algorithm implemented in *ToASTD* program^73^, available at http://nmr.cent3.uw.edu.pl. *CcpNmr* Analysis was employed to analyze the spectra^74^.

For analyzing titration data, shift changes between ^1^H-^15^N TROSY spectra of ID5_F1 at different concentrations and with added IQGAP1-F were calculated as average Euclidean distances using the formula:

$$\sqrt{[({1/2}^{\ast }(({\Delta }^{{\prime\prime} }{{\rm{1H}}}^{{\prime\prime} })]^2+[({{\rm{\alpha }}}^{\ast }{\Delta }^{{\prime\prime} }15{{\rm{N}}}^{{\prime\prime} })]^2))}$$

using a weighting factor 0.14, and in case of glycines, 0.2.

### Circular dichroism (CD) spectroscopy

Far-UV CD spectra ^of ID5 (26 µM) and IQGAP1-F (21 µM) were recorded on a J-715 spectropolarimeter in 50 mM KH_2_PO_4_, 150 mM NaCl buffer pH 6.5. Data points were collected every 0.5 nm at the controlled temperature of 24 °C. CD data were fitted using DichroWeb^24^ with CDSSTR data sets 4 and 7 for ID5 and IQGAP1-F, respectively.

### Bio-layer interferometry assay

To measure the affinity between IQGAP1 and ID5, the association and dissociation phases were recorded for 60 seconds each for twelve different concentrations were measured. To this end, GST-tagged ID5 was immobilized in the surface of the biosensors and the BLI signal at a series of IQGAP1-F concentrations spanning from 0.1 to 19 µM was measured. All steps were performed at RT. The sensorgrams were double referenced against the buffer reference signal and the nonspecific binding (GST) by the Data Analysis software 9.0 (ForteBio). The steady-state was fitted using Prism7 software with a 1∶1 binding kinetics.

### Pull-down assays

#### ID5 fragments with IQGAP1-F interaction assessed by pull-down assay

A series of pull-down assays was performed using ID5 fragments (F1, F2 and F3) solubilized in PBS. His-tagged IQGAP1-F was biotinylated (EZ-Link Sulfo-NHS-Biotinylation kit, Thermo Scientific) and immobilized for one hour on Streptavidin magnetic beads (GE Healthcare). The loaded beads were blocked for 15 min with PBS containing 0.1% Tween and 5% milk powder before washing them twice with PBS. Then, 100 µl beads were incubated for 1 h with 300 µl of either purified His-tagged ID5_F1, ID5_F2 or ID5_F3 (concentration 30 µM). Before eluting the proteins, the beads were washed three times with PBS. Then, the first elution step was performed using 100 µl elution buffer (50 mM sodium phosphate, 100 mM NaCl, 2% SDS, 2 M Urea). The beads were incubated in elution buffer for 5 min at 98 °C. The supernatant was removed and loaded on an SDS-PAGE after addition of loading dye. For the second elution step, the beads were incubated in 30 µl 1x SDS loading dye for 5 min at 98 °C. The supernatant was directly loaded on a gel. The gels were further analyzed by Western blot using anti-His antibodies.

### ID5 with IQGAP1-F interaction assessed by pull-down assay

As a control, we also performed pull-down assays with immobilized full ID5 and IQGAP1-F. His-tagged ID5 was biotinylated (EZ-Link Sulfo-NHS-Biotinylation kit, Thermo Scientific) and immobilized for one hour on Streptavidin magnetic beads (GE Healthcare). The pull-down assay was performed as described above using 20 µM IQGAP1-F in PBS.

### Acetylation assay

*In vitro* acetylation assays were performed in 50 μl buffer (50 mM NaH_2_PO_4_, 125 mM NaCl, 0.5 mM DTT, pH 7.4) for 30 min at 30 °C. For the reaction, 624 pmols of lyophilized histone 4 (Millipore or Sigma-Aldrich) was acetylated with 9 pmols HAT-active full-length CBP or CBP core domain, or the HAT domain of PCAF (Cayman chemical, No. 10009115) or KAT5 (Sanbio, No. 10783–100). As a coenzyme in the acetylation reaction, 20 µmols acetyl-CoA was added. In some cases, IQGAP1 or ID5 was added to the reaction in 10 or 100-fold excess relative to CBP. The samples were analyzed by Western blot by an anti-acetylated-lysine (Bioke 9814S) and anti-His antibodies.

### Phylogenetic tree construction

Experimental evidence suggested that IQGAP1-F interacts with the HAT domains of CBP/P300, PCAF and KAT5, albeit these interactions have markedly different functional consequences for KAT5 than the others. We investigated the phylogenetic relations of various acetyl-transferase domain families in order to evaluate whether the difference in behaviour could be attributed to long evolutionary distances. According to Pfam^75^, the acetyltransferase domain of CBP/P300 belongs to the HAT_KAT11 domain family (Pfam ID PF08214), while the domain of PCAF is of the Acetyltransf_1 family (Pfam ID PF00583) and for KAT5 it is MOZ_SAS (Pfam ID PF01853). All three domain families belong to the same domain clan, called Acetyltrans (Pfam ID CL0257). We have selected all the available human proteins in the clan, encompassing 113 proteins from 39 different domain families (out of 146995 sequences overall). After retrieving these domain sequences, we have performed multiple sequence alignment using Clustal Omega^76^ saving the output as a Neighbour-joining tree without distance corrections. The tree was then displayed and annotated using the webserver iTOL (Interactive Tree of Life)^77^.

## Supplementary information

Supplementary Information
