# Supplementary material for: Interaction between the scaffold proteins CBP by IQGAP1 provides an interface between gene expression and cytoskeletal activity
Source: Sci Rep. 2020 Apr 1;10:5753. doi: 10.1038/s41598-020-62069-w (PMC7113243; doi:10.1038/s41598-020-62069-w)
Supplement: Supplementary file 1 — Supplementary Information [file 41598_2020_62069_MOESM1_ESM.docx]

**Interaction between the scaffold proteins CBP by IQGAP1 provides an interface between gene expression and cytoskeletal activity**

*Simone Kosol, Sara Contreras-Martos, Alessandro Piai, Mihaly Varadi, Tamas Lazar, Angela Bekesi, Pierre Lebrun, Isabella C. Felli, Roberta Pierattelli and Peter Tompa*

**Supplementary information**

**Supplementary Figures**

**
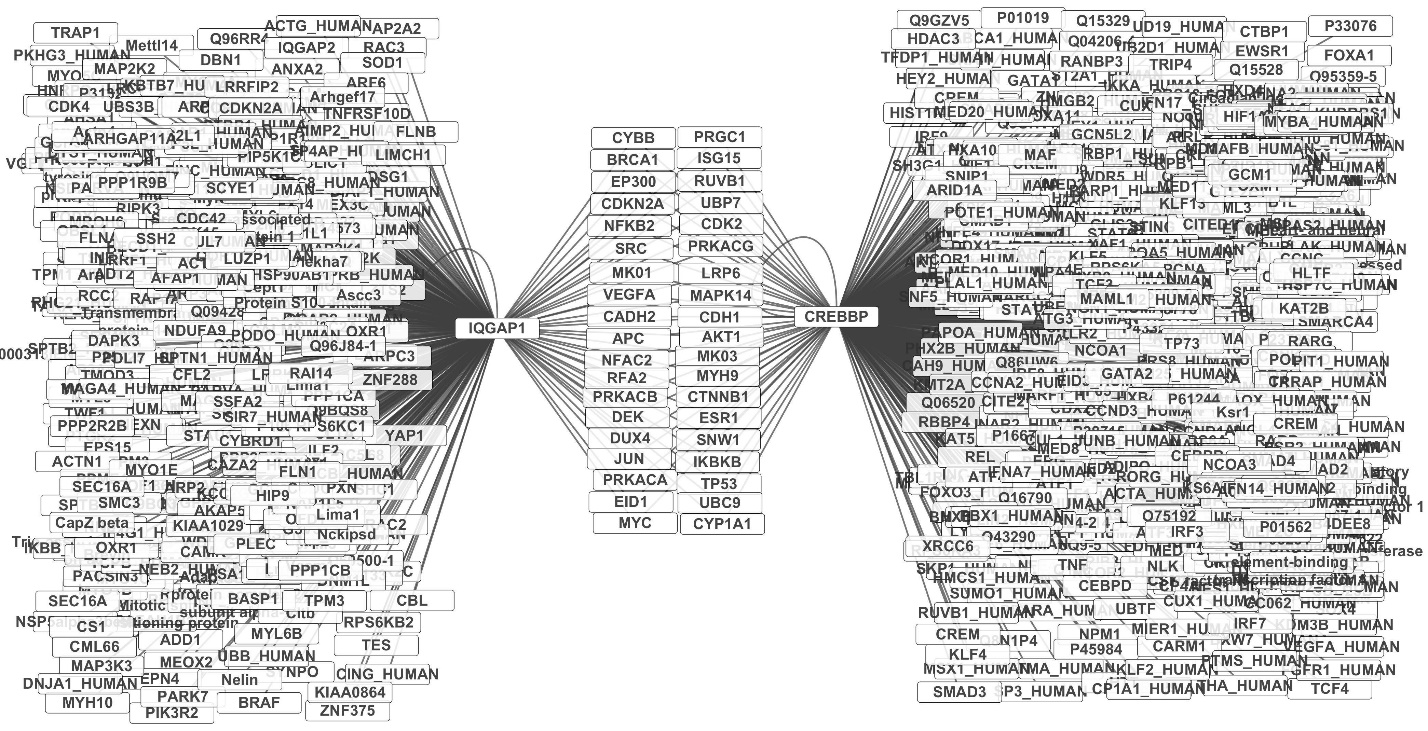
**

**Figure S1** *Interaction partners of CBP and IQGAP1*

Interaction partners of CBP (UniProt Q92793) and IQGAP1 (UniProt P46940) were retrieved by database search with their UniProt accessions in Cytoscape 3.5 that has access to 34 interaction databases. For the human CBP and IQGAP1, 950 and 627 interaction partners were found, which were restricted to protein-protein interactions (PPI) and then cross-species interactions were filtered out by selecting only human (taxonomy ID: 9606) proteins. CBP and IQGAP had 810 and 564 human PPIs respectively, of which 38 were found to interact with both of them.





**Figure S2** *Results of yeast-two-hybrid assays*

ID5 was used as a bait in Y2H analysis of human placenta- and fetal-brain libraries. (a) Significant hits were found in the fetal brain and human placenta libraries with three proteins detected in both. A complete list can be found in Suppl. Table S1. (b) GO annotated functions of interaction partners identified by yeast-two-hybrid assays and number of acetylated proteins from each category (orange) as listed in CPLM, the database of protein lysine modifications ^1^.

**

**

**Figure S3** *Pull-down analysis of the interaction of ID5 and IQGAP1-F*

a) IQGAP1-F (left; aa 286-592, encompassing its CBP-binding region, cf. Figure 1) and ID5 (right) were biotinylated and immobilized on magnetic streptavidin beads and incubated with ID5 and IQGAP1-F, respectively. The beads were washed and the eluate was analyzed by Western blot using anti-His antibodies. b) In control experiments IQGAP1-F (left) and ID5 (right) were incubated with streptavidin beads without biotinylated proteins and eluted as before. c) Pull-down assays with immobilized biotinylated IQGAP1-F and ID5-F1 (left, see also figure 5), ID5-shortF2 (center) and ID5-F3 (right). IQGAP1-F is also present in the elution fractions which were analyzed by Western blots using anti-His antibodies.


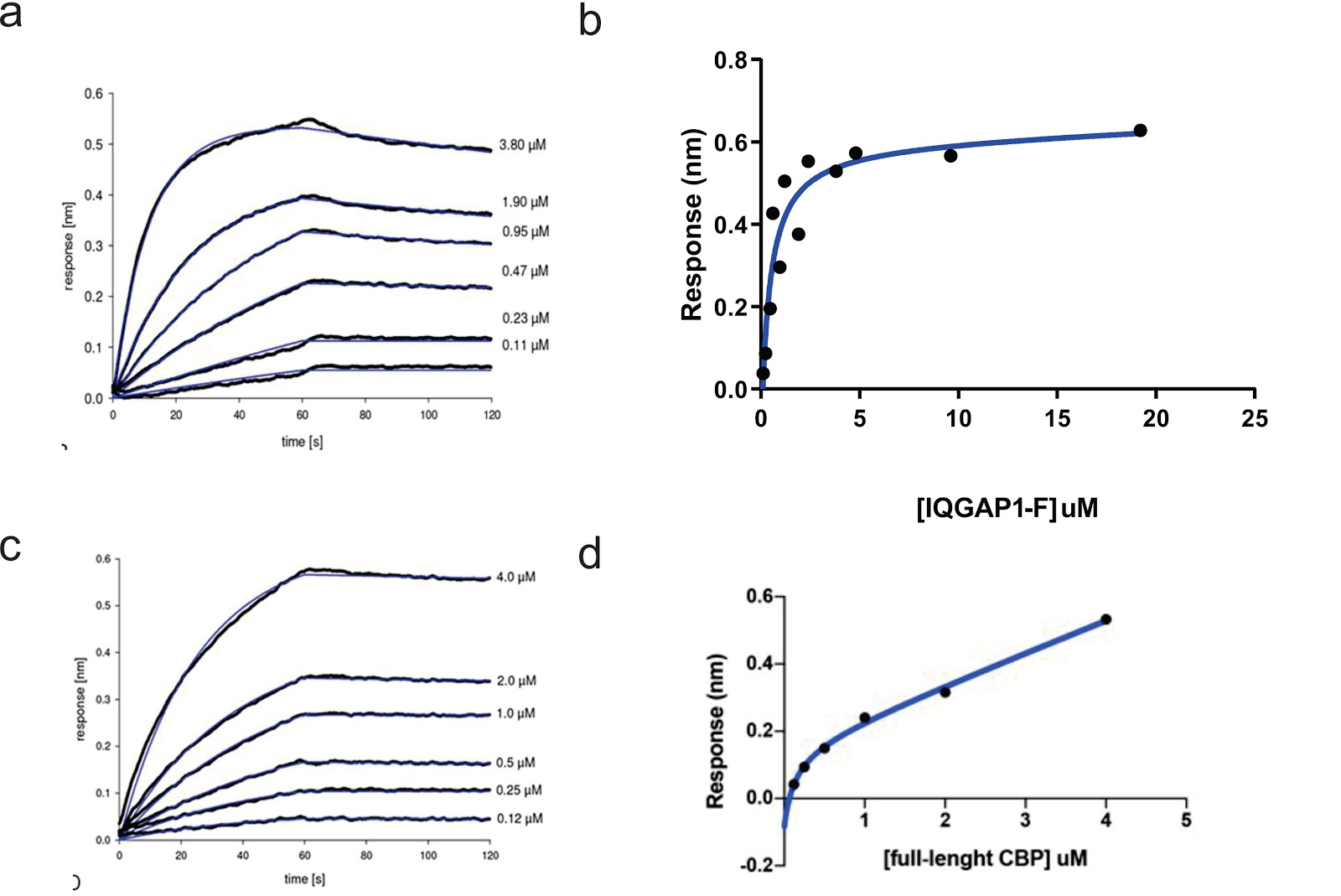


**Figure S4** *Interaction of CBP with IQGAP characterized by biolayer interferometry (BLI)*

The Kd of the interaction of IQGAP1-F with ID5 (a,b) and with full-length CBP (c,d) has been determined by BLI. We immobilized either GST-tagged ID5 and titrated it with IQGAP1-F, or His-tagged IQGAP1-F, and titrated it with full-length CBP. Sensorgrams were fitted separately by a 1:1 binding model and steady-state analysis suggest a Kd in the low μM range (Kd = 0.4 ± 0.3 μM) for ID5-IQGAP1-F and ~0.4 μM for CBP-IQGAP1-F (it is to be noted that titration with full-length CBP does not reach saturation, which suggests a more complex binding mechanism, i.e. the resulting Kd can only be considered as an approximation). Fitting quality is R2>0.88 of the steady-state.


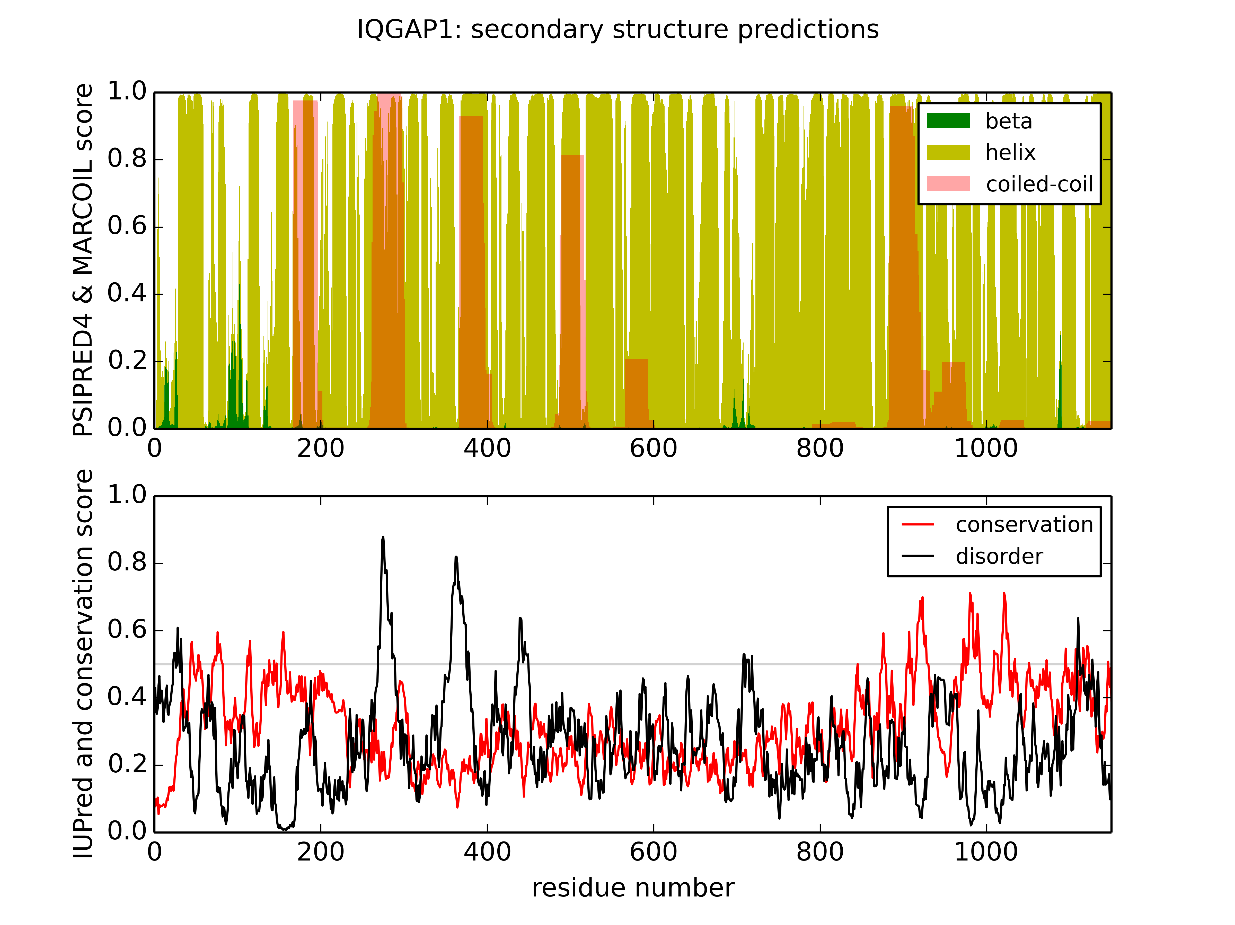


**Figure S5** *Sequence-based prediction results for IQGAP*

Secondary structure was predicted to IQGAP1 (UniProt P46940, aa. 1-650)by PSIPRED 4.0, followed by coiled coil prediction in MARCOIL ^2^ with default parameters and long disorder prediction in IUPred 2 ^3^. For all predictors, an amino acid score of 1 means that the residue is located in a regions presenting the given feature, while score of 0 means the lack of the feature is predicted. A simplified conservation score was computed using an ortholog alignment (KOG2128, 385 proteins) from the eggNOG 4.5.1 database by calculating the fraction of residue positions in the alignment identical to the human IQGAP1. The CBP-binding domain (CBD) is found between amino acid positions 346 and 556.


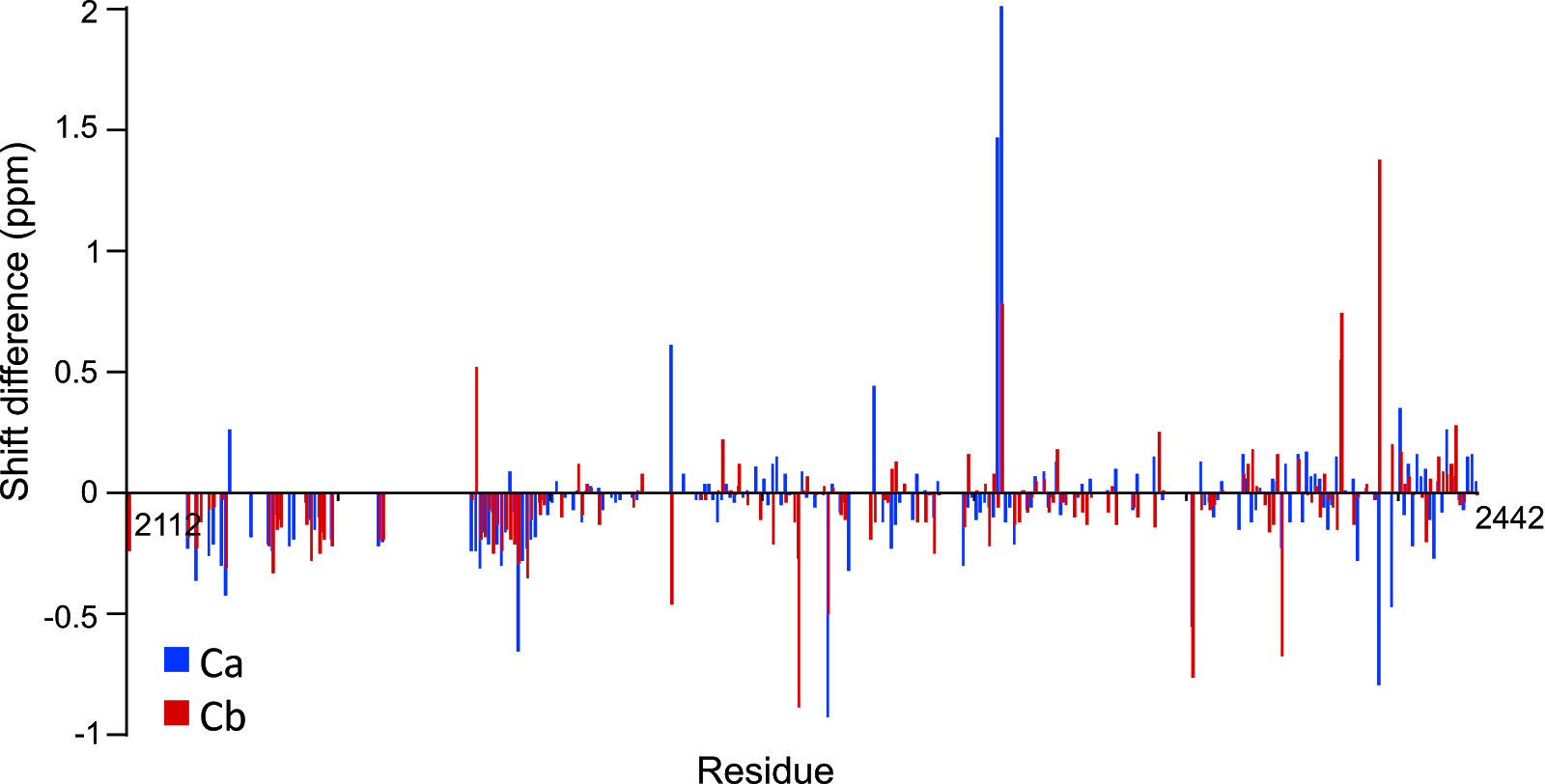


**Figure S6** *Comparison of chemical shifts of ID5 and its fragments*

C^𝛼^ (Ca, blue) and C^𝛽^ (Cb, red) chemical shifts have been determined for all residues in full-length ID5, and for the same residues in its three fragments, ID5_F1, ID5_F2 and ID5_F3 (cf. Suppl. Figure S1). Calculated differences between the values for full-length ID5 and its fragments are small throughout the sequence, suggesting that the residues occur in very similar structural environment, i.e. dividing the protein into shorter fragments has little – if any – local structural effect.

**
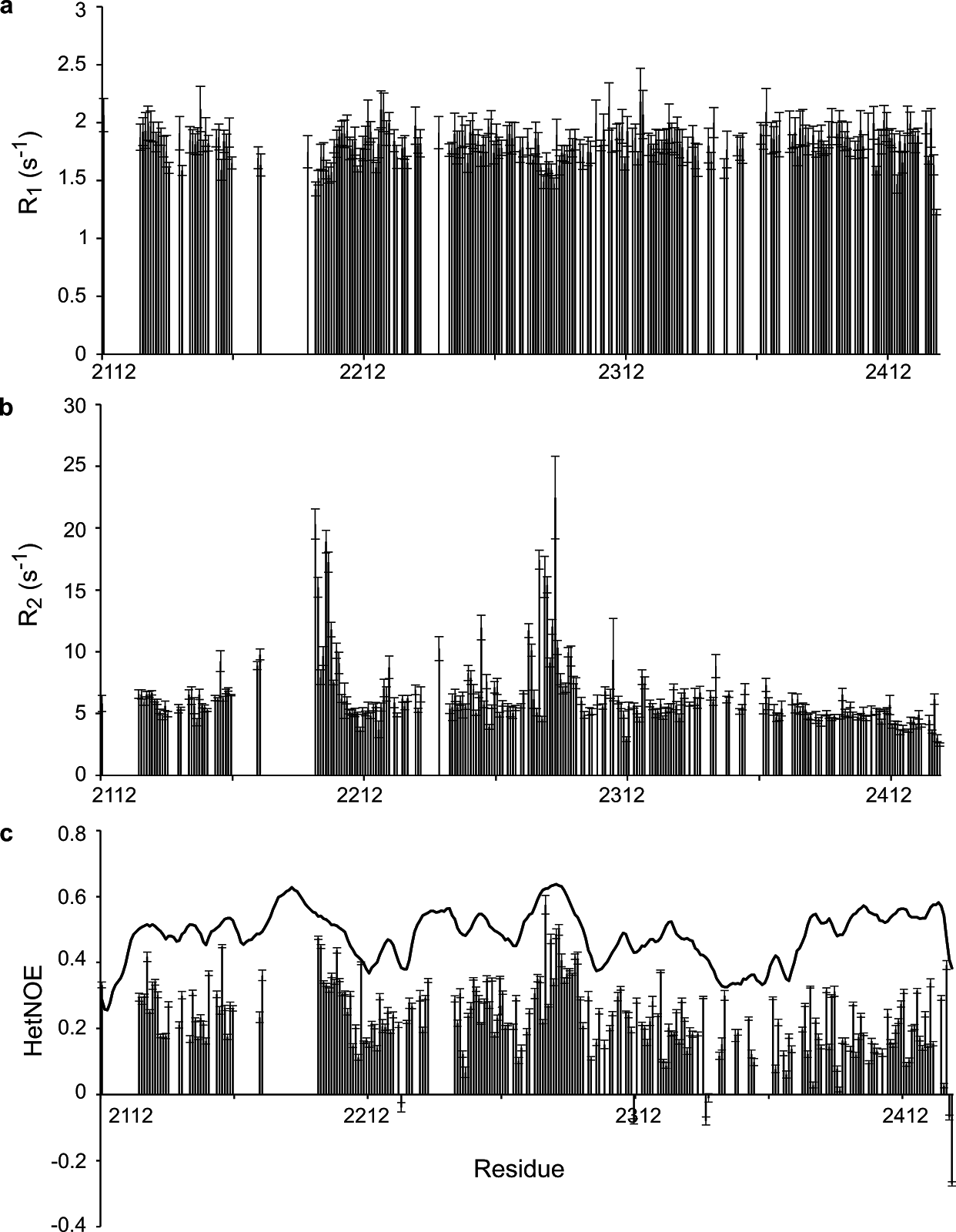
**

**Figure S7** *NMR relaxation parameters of ID5*

Three relaxation parameters, ^15^N R_1_ (a) and R_2_ (b) rates and HetNOE values (c) have been determined for ID5. For a comparison, the black line overlaid on HetNOE values (c) represents an S^2^-value predicted by DynaMine ^4, 5^.


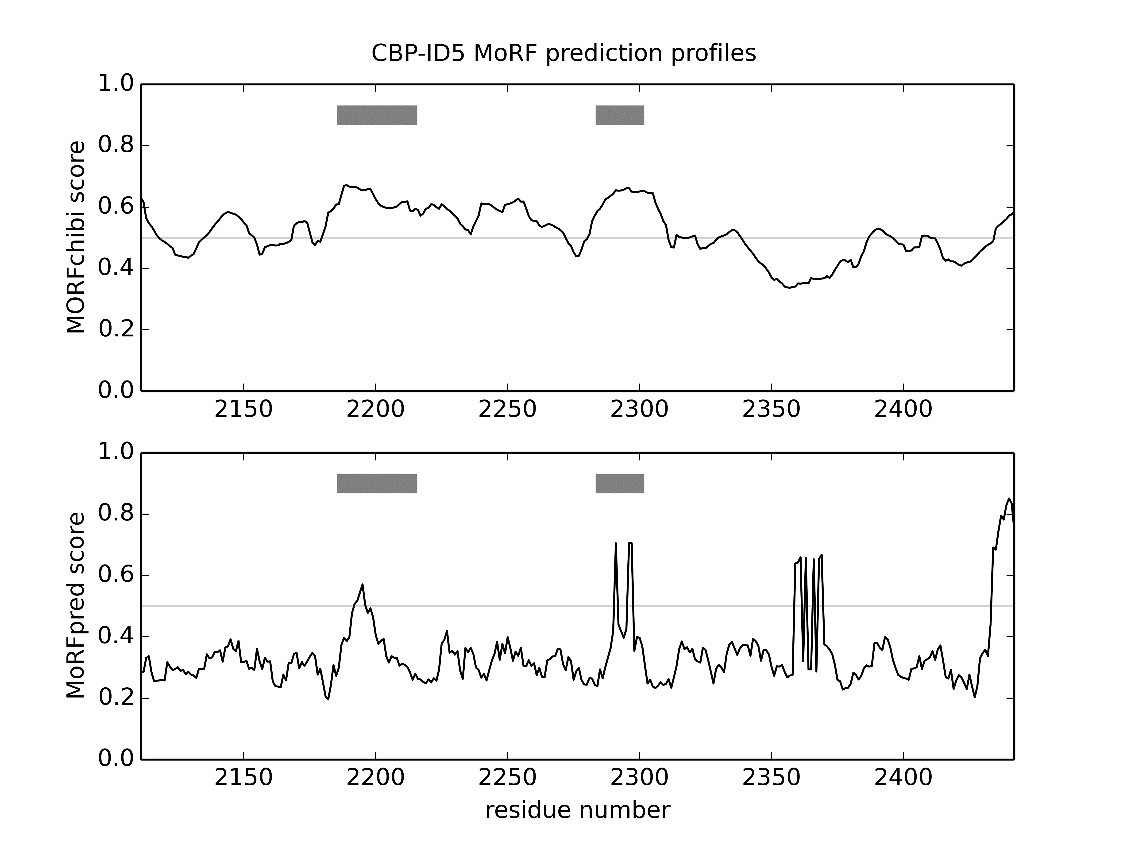


**Figure S8** *Protein-protein interaction sites in ID5 by MoRF prediction*

Molecular recognition features (MoRFs) were predicted for CBP-ID5 using the MoRFchibi ^6^ and MoRFpred ^7^ tools. For both predictors, an amino acid score of 1 means that the residue is predicted to be located in a MoRF, while score of 0 means the lack of the feature is predicted. Usually, a cutoff value of 0.5 is used to discretize the prediction scores to positives and negatives. Helix 1 and helix 2 regions identified by NMR were shown as gray boxes.


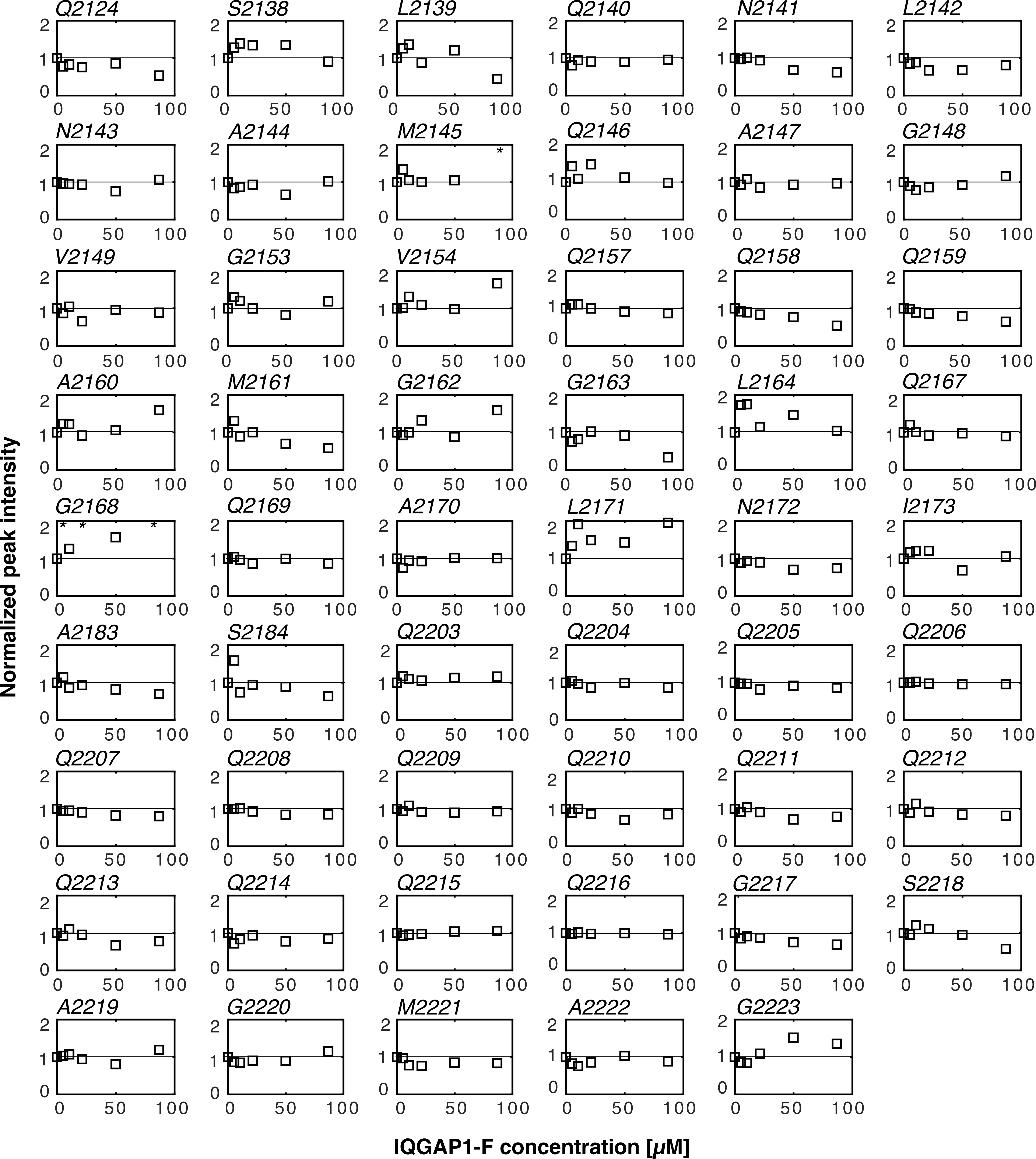


**Figure S9** *Changes in peak volumes during titrations of ID5-F1 with IQGAP1-F.*

Peak volumes were normalized to the peak volume of the first titration point (with no IQGAP1-F added). Titration points that exceed a normalized peak volume of 2 are indicated with an asterisk (*). Only residues that could be assigned in the ^15^N-HSQC spectrum of ID5-F1 are shown.


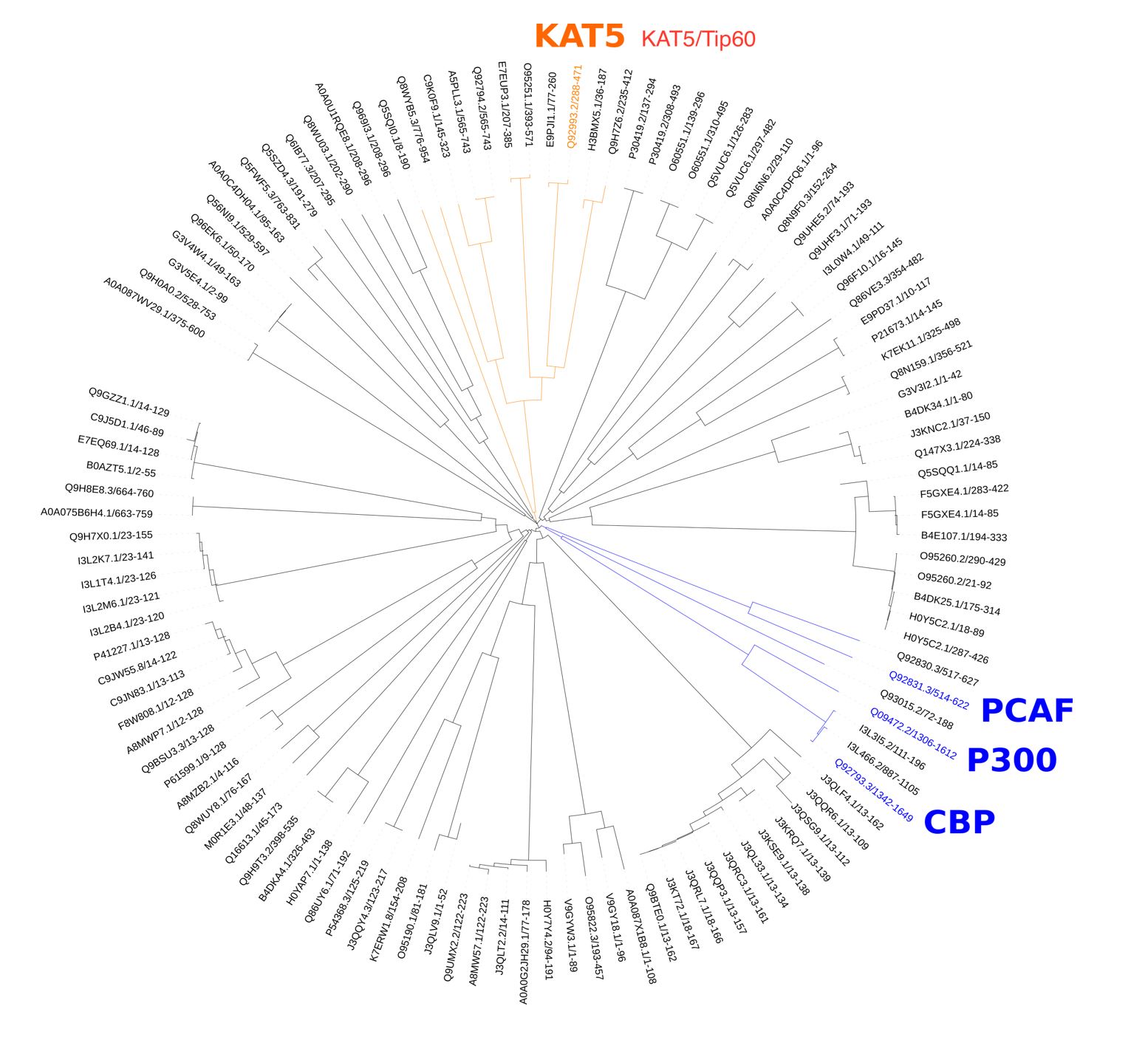


**Figure S10** *Phylogenetic tree of the HAT family*

Distance tree based on sequence similarity of various human protein domains belonging to the acetyltransferase domain clan. According to the comparative analysis, the acetyl- transferase domains of CBP, P300 and PCAF are highly similar (blue, belonging to the family KAT3), while KAT5 (orange) is significantly different, belonging to another cluster of domain families. This difference in sequence might lead to the observed difference in interactions with IQGAP1.

^
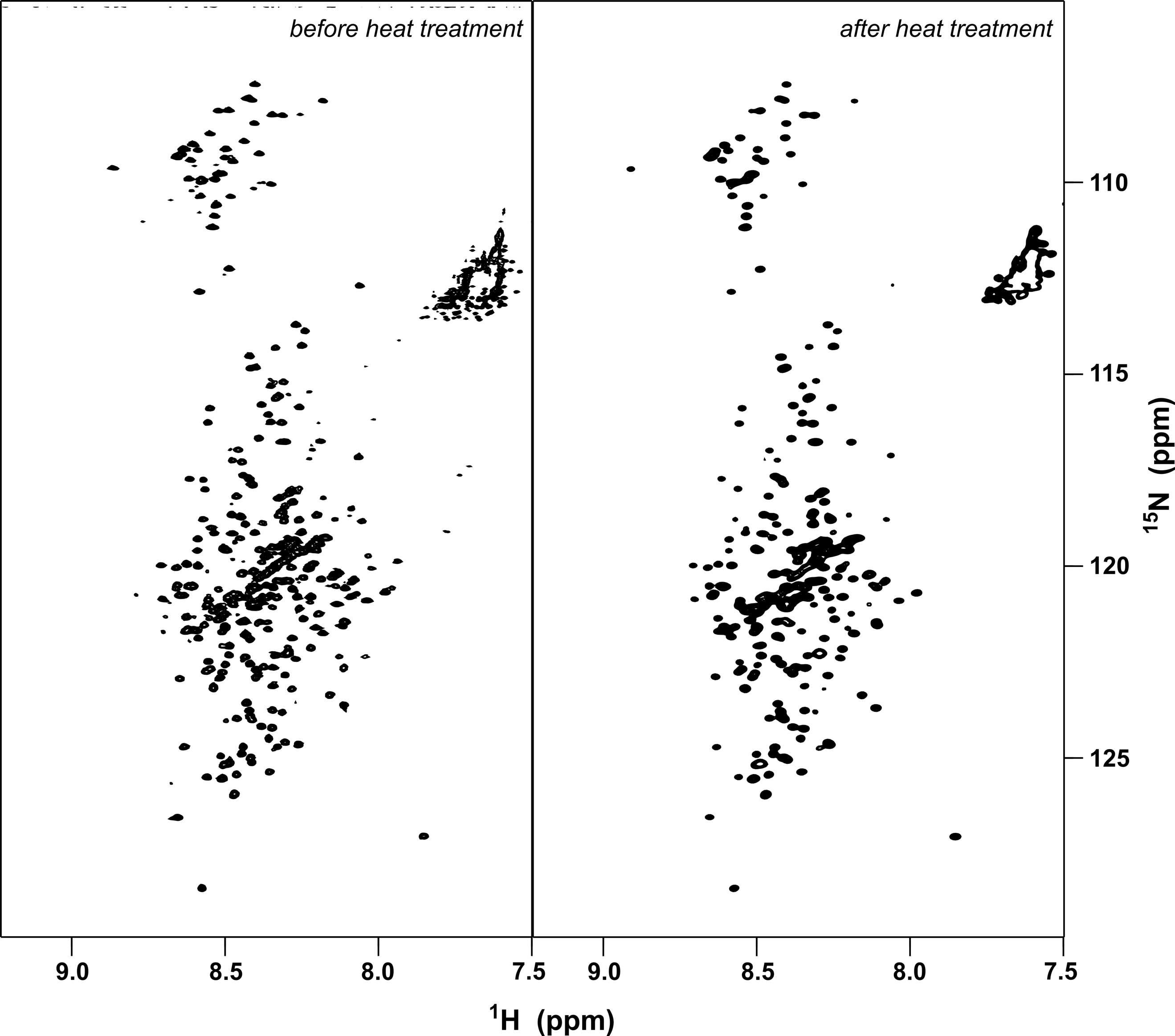
^

**Figure S11** *Lack of structural effect on ID5 of boiling*

^15^N-TROSY-HSQC spectrum of ID5 recorded before heat treatment (left) and ^15^N-HSQC spectrum recorded after boiling for ten minutes at 85℃ (right). The spectra were acquired at 900 MHz and 700 MHz, respectively.

**Supplementary Tables**

**Table S1** Potential cellular interaction partners of CBP ID5

Interaction partners have been observed by Y2H analysis in two libraries (placenta and fetal brain). UniProt ID of the partner, its GO “molecular function” (MF) term and the number of hits for the given protein in the Y2H screen are also shown.

| **Interaction partner** | **Uniprot ID** | **Origin cDNA library** | **Hits in Y2H** | **GO molecular function** |
| --- | --- | --- | --- | --- |
| Acetyl-coenzyme A synthetase 2-like, mitochondrial | F5H6F4 | Placenta | 1 | acetate-CoA ligase activity |
| Actin, cytoplasmic 1 | P60709 | Fetal Brain | 1 | structural constituent of cytoskeleton |
| ANKS1A | Q92625 | Placenta | 1 | ephrin receptor binding |
| ANKS1Bvar7 | Q7Z6G8 | Fetal Brain | 1 | ephrin receptor binding |
| ANKS1Bvar2 | Q7Z6G8-3 | Fetal Brain | 2 | ephrin receptor binding |
| ANKS1Bvar9 | Q7Z6G8-2 | Fetal Brain | 5 | ephrin receptor binding |
| Annexin A7 | P20073 | Fetal Brain | 1 | RNA binding |
| Amyloid beta A4 protein | P05067 | Fetal Brain | 1 | acetylcholine receptor binding |
| BAG family molecular chaperone regulator 5 | Q9UL15 | Fetal Brain | 1 | adenyl-nucleotide exchange factor activity |
| Tubulin beta chain | P07437 | Fetal Brain | 1 | GTPase activating protein binding |
| Centriolin | Q7Z7A1 | Fetal Brain | 1 | protein tyrosine kinase activity |
| Centrosomal protein of 290 kDa | O15078 | Fetal Brain | 1 | identical protein binding |
| Chordin-like protein 1 | Q9BU40 | Fetal Brain | 1 |  |
| CLIP-associating protein 2 | O75122 | Fetal Brain | 1 | actin filament binding |
| Elongation factor 1-alpha 1 | P68104 | Fetal Brain | 1 | GTPase activity |
| Elongation factor 2 | P13639 | Fetal Brain | 1 | actin filament binding |
| Eukaryotic translation initiation factor 4 gamma 2 | P78344 | Fetal Brain | 1 | cadherin binding |
| RNA-binding protein EWS | Q01844 | Placenta | 1 | calmodulin binding |
| RNA-binding protein FUS | P35637 | Fetal Brain | 1 | estrogen receptor binding |
| Lysosomal alpha-glucosidase | P10253 | Placenta | 2 | alpha-1,4-glucosidase activity |
| Transcriptional activator GLI3 | P10071 | Fetal Brain | 1 | beta-catenin binding |
| G-protein coupled receptor 161 | Q8N6U8 | Fetal Brain | 1 | G-protein coupled receptor activity |
| Glutamate receptor 3 | P42263 | Fetal Brain | 1 | AMPA glutamate receptor activity |
| Glutathione S-transferase omega-2 | Q9H4Y5 | Placenta | 2 | glutathione transferase activity |
| Beta-hexosaminidase subunit alpha | P06865 | Placenta | 1 | acetylglucosaminyltransferase activity |
| Zinc finger protein 40 | P15822 | Placenta | 1 | DNA binding |
| Transcription factor HIVEP2 | P31629 | Fetal Brain | 6 | DNA binding |
| High mobility group protein 20A | Q9NP66 | Placenta, Fetal Brain | 1, 5 | DNA binding |
| Heterogeneous nuclear ribonucleoprotein L | P14866 | Fetal Brain | 1 | RNA binding |
| E3 ubiquitin-protein ligase HUWE1 | Q7Z6Z7 | Fetal Brain | 1 | DNA binding |
| Ras GTPase-activating-like protein IQGAP1 | P46940 | Placenta | 5 | cadherin binding |
| Lysine-specific demethylase 6B | O15054 | Fetal Brain | 1 | beta-catenin binding |
| Lysine-specific demethylase 2A | Q9Y2K7 | Fetal Brain | 2 | DNA binding |
| KH domain-containing, RNA-binding, signal transduction-associated protein 1 | Q07666 | Fetal Brain | 1 | DNA binding |
| Calmodulin-regulated spectrin-associated protein 2 | Q08AD1 | Placenta, Fetal Brain | 1, 6 | calmodulin binding |
| Ectopic P granules protein 5 homolog | Q9HCE0 | Fetal Brain | 1 |  |
| Kinesin heavy chain isoform 5A | Q12840 | Fetal Brain | 1 | microtubule binding |
| Microtubule-associated protein 1B | P46821 | Fetal Brain | 1 (3) | microtubule binding |
| Unconventional myosin-Ie | Q12965 | Fetal Brain | 1 | actin filament binding |
| UDP-N-acetylglucosamine--peptide N-acetylglucosaminyltransferase 110 kDa subunit | O15294 | Fetal Brain | 1 | acetylglucosaminyltransferase activity |
| Pappalysin-1 | Q13219 | Placenta | 2 | metalloendopeptidase activity |
| Paired box protein Pax-6 | P26367 | Fetal Brain | 1 | DNA binding |
| Pleckstrin homology-like domain family A member 1 | Q8WV24 | Fetal Brain | 1 |  |
| DNA repair protein RAD50 | Q92878 | Fetal Brain | 1 | DNA binding |
| RNA-binding protein 24 | Q9BX46 | Fetal Brain | 1 | mRNA 3'-UTR binding |
| RNA-binding protein 5 | P52756 | Fetal Brain, Placenta | 1, 1 | DNA binding |
| Riboflavin kinase | Q969G6 | Placenta | 1 | ATP binding |
| RING-box protein 2 | Q9UBF6 | Fetal Brain | 1 | copper ion binding |
| 40S ribosomal protein S20 | P60866 | Fetal Brain | 1 | RNA binding |
| Exocyst complex component 5 | O00471 | Fetal Brain | 1 | Ral GTPase binding |
| Semaphorin-3B | Q13214 | Placenta | 1 | axon guidance |
| Neuronal-specific septin-3 | Q9UH03 | Fetal Brain | 1 | GTP binding |
| Neutral amino acid transporter A | P43007 | Fetal Brain | 1 | amino acid transmembrane transporter activity |
| snRNA-activating protein complex subunit 5 | O75971 | Fetal Brain | 1 | transcription factor activity |
| U5 small nuclear ribonucleoprotein 200 kDa helicase | O75643 | Fetal Brain | 1 | ATP-dependent helicase activity |
| Son of sevenless homolog 2 | Q07890 | Fetal Brain | 1 | DNA binding |
| Spectrin alpha chain, non-erythrocytic 1 | Q13813 | Fetal Brain | 1 | actin binding |
| Synaptotagmin-1 | P21579 | Fetal Brain | 2 | calcium-dependent protein binding |
| Nucleolysin TIAR | Q01085 | Placenta | 1 | DNA binding |
| T-lymphoma invasion and metastasis-inducing protein 1 | Q13009 | Fetal Brain | 2 | microtubule binding |
| Apoptosis-stimulating of p53 protein 2 | Q13625 | Fetal Brain | 1 | p53 binding |
| Lysine-specific demethylase 6A | O15550 | Fetal Brain | 1 | chromatin DNA binding |
| Synaptic vesicle membrane protein VAT-1 homolog-like | Q9HCJ6 | Fetal Brain | 1 | oxidoreductase activity |
| Wiskott-Aldrich syndrome protein | P42768 | Placenta | 2 | actin binding |
| YTH domain-containing family protein 2 | Q9Y5A9 | Placenta | 1 | RNA binding |

**Table S2** Assigned chemical shifts of ID5 and ID5 fragments in ppm.

|  |  | **ID5** | | | | | **ID5_F1** | | | | | **ID5_F2** | | | | | **ID5_F3** | | | | |
| --- | --- | --- | --- | --- | --- | --- | --- | --- | --- | --- | --- | --- | --- | --- | --- | --- | --- | --- | --- | --- | --- |
| **Residue Number** | **Amino acid** | ***H_N_*** | ***N*** | ***C'*** | ***Cα*** | ***Cβ*** | ***H_N_*** | ***N*** | ***C'*** | ***Cα*** | ***Cβ*** | ***H_N_*** | ***N*** | ***C'*** | ***Cα*** | ***Cβ*** | ***H_N_*** | ***N*** | ***C'*** | ***Cα*** | ***Cβ*** |
| 2124 | Q | 8.5 | 122.89 |  | 53.72 | 28.97 | 8.45 | 122.84 | 174.02 | 53.95 | 29.21 |  |  |  |  |  |  |  |  |  |  |
| 2125 | P |  |  |  |  |  |  |  |  |  |  |  |  |  |  |  |  |  |  |  |  |
| 2126 | G |  |  |  |  |  |  |  |  |  |  |  |  |  |  |  |  |  |  |  |  |
| 2127 | M |  |  |  |  |  |  |  |  |  |  |  |  |  |  |  |  |  |  |  |  |
| 2128 | Q |  |  |  |  |  |  |  |  |  |  |  |  |  |  |  |  |  |  |  |  |
| 2129 | P |  |  |  |  |  |  |  |  |  |  |  |  |  |  |  |  |  |  |  |  |
| 2130 | Q |  |  |  |  |  |  |  |  |  |  |  |  |  |  |  |  |  |  |  |  |
| 2131 | P |  |  |  |  |  |  |  |  |  |  |  |  |  |  |  |  |  |  |  |  |
| 2132 | G |  |  |  |  |  |  |  |  |  |  |  |  |  |  |  |  |  |  |  |  |
| 2133 | M |  |  |  |  |  |  |  |  |  |  |  |  |  |  |  |  |  |  |  |  |
| 2134 | H |  |  |  |  |  |  |  |  |  |  |  |  |  |  |  |  |  |  |  |  |
| 2135 | Q |  |  |  |  |  |  |  |  |  |  |  |  |  |  |  |  |  |  |  |  |
| 2136 | Q |  |  |  |  |  |  |  |  |  |  |  |  |  |  |  |  |  |  |  |  |
| 2137 | P |  |  | 176.84 |  |  |  |  | 176.91 | 63.61 | 32.32 |  |  |  |  |  |  |  |  |  |  |
| 2138 | S | 8.55 | 116.35 | 174.88 | 58.24 | 63.86 | 8.57 | 116.34 | 174.94 | 58.47 | 64.04 |  |  |  |  |  |  |  |  |  |  |
| 2139 | L | 8.47 | 124.42 |  |  |  | 8.48 | 124.34 |  |  |  |  |  |  |  |  |  |  |  |  |  |
| 2140 | Q | 8.37 | 120.16 | 175.85 | 56.21 | 29.43 | 8.38 | 120.13 | 175.94 | 56.57 | 29.66 |  |  |  |  |  |  |  |  |  |  |
| 2141 | N | 8.45 | 119.68 | 175.59 | 53.48 | 38.65 | 8.46 | 119.63 | 175.64 |  | 38.77 |  |  |  |  |  |  |  |  |  |  |
| 2142 | L | 8.37 | 123.03 | 177.72 | 56.26 | 42.03 | 8.39 | 123.01 |  |  |  |  |  |  |  |  |  |  |  |  |  |
| 2143 | N | 8.46 | 118.59 | 175.5 | 53.61 | 38.69 |  |  | 175.51 | 53.87 | 38.76 |  |  |  |  |  |  |  |  |  |  |
| 2144 | A | 8.15 | 123.74 | 182.13 | 53.2 | 19.23 | 8.16 | 123.75 | 178.18 | 53.41 | 19.29 |  |  |  |  |  |  |  |  |  |  |
| 2145 | M | 8.27 | 118.7 | 180.63 | 55.95 | 32.75 | 8.29 | 118.73 | 176.66 |  |  |  |  |  |  |  |  |  |  |  |  |
| 2146 | Q | 8.33 | 121.24 | 175.79 | 55.79 | 29.6 | 8.34 | 121.25 | 175.82 | 56.09 | 29.63 |  |  |  |  |  |  |  |  |  |  |
| 2147 | A | 8.41 | 125.38 | 178.25 | 52.76 | 19.12 | 8.43 | 125.38 | 178.27 | 53.18 | 19.43 |  |  |  |  |  |  |  |  |  |  |
| 2148 | G | 8.42 | 108.15 | 173.87 | 45.54 |  | 8.45 | 108.21 | 173.84 | 45.28 |  |  |  |  |  |  |  |  |  |  |  |
| 2149 | V | 7.97 | 121.05 | 174.57 | 60.01 | 32.59 | 8 | 121.11 |  |  |  |  |  |  |  |  |  |  |  |  |  |
| 2150 | P |  |  |  |  |  |  |  |  |  |  |  |  |  |  |  |  |  |  |  |  |
| 2151 | R |  |  |  |  |  |  |  |  |  |  |  |  |  |  |  |  |  |  |  |  |
| 2152 | P |  |  | 177.6 |  |  |  |  | 177.61 |  |  |  |  |  |  |  |  |  |  |  |  |
| 2153 | G | 8.6 | 109.76 | 173.83 | 45.16 |  | 8.63 | 109.83 | 173.84 | 45.34 |  |  |  |  |  |  |  |  |  |  |  |
| 2154 | V | 8.03 | 121.26 | 174.26 | 59.7 | 32.62 | 8.05 | 121.32 |  |  |  |  |  |  |  |  |  |  |  |  |  |
| 2155 | P |  |  |  |  |  |  |  |  |  |  |  |  |  |  |  |  |  |  |  |  |
| 2156 | P |  |  | 177.19 |  |  |  |  | 177.19 | 63.47 | 32.26 |  |  |  |  |  |  |  |  |  |  |
| 2157 | Q | 8.62 | 120.32 | 176.3 | 56.12 | 29.33 | 8.64 | 120.35 | 176.31 | 56.33 | 29.55 |  |  |  |  |  |  |  |  |  |  |
| 2158 | Q | 8.51 | 121.75 | 176.05 | 56.02 | 29.43 | 8.53 | 121.76 | 176.08 | 56.26 | 29.76 |  |  |  |  |  |  |  |  |  |  |
| 2159 | Q | 8.5 | 122.08 | 175.82 | 56.09 | 29.56 | 8.52 | 122.1 | 175.82 | 56.18 | 29.71 |  |  |  |  |  |  |  |  |  |  |
| 2160 | A | 8.5 | 125.54 |  | 52.74 | 19.05 | 8.52 | 125.55 | 177.96 | 52.85 | 19.19 |  |  |  |  |  |  |  |  |  |  |
| 2161 | M |  |  | 176.77 |  |  | 8.51 | 119.97 | 177.01 |  |  |  |  |  |  |  |  |  |  |  |  |
| 2162 | G | 8.52 | 110.23 | 174.6 | 45.43 |  | 8.52 | 110.19 | 174.61 | 45.65 |  |  |  |  |  |  |  |  |  |  |  |
| 2163 | G | 8.34 | 108.57 | 173.97 | 45.17 |  | 8.36 | 108.61 | 173.99 | 45.36 |  |  |  |  |  |  |  |  |  |  |  |
| 2164 | L | 8.21 | 121.16 |  |  |  | 8.23 | 121.15 |  |  |  |  |  |  |  |  |  |  |  |  |  |
| 2165 | N |  |  |  |  |  |  |  |  |  |  |  |  |  |  |  |  |  |  |  |  |
| 2166 | P |  |  | 177.36 | 64.2 | 32.18 |  |  | 177.35 | 64.24 | 32.31 |  |  |  |  |  |  |  |  |  |  |
| 2167 | Q | 8.45 | 119.1 | 176.78 | 56.22 | 29.15 | 8.46 | 119.07 | 176.77 | 56.33 | 29.43 |  |  |  |  |  |  |  |  |  |  |
| 2168 | G | 8.38 | 109.58 | 174.24 | 45.68 |  | 8.4 | 109.64 | 174.22 | 45.83 |  |  |  |  |  |  |  |  |  |  |  |
| 2169 | Q | 8.24 | 119.99 | 175.86 | 55.72 | 29.58 | 8.25 | 119.89 | 175.82 | 55.82 | 29.83 |  |  |  |  |  |  |  |  |  |  |
| 2170 | A | 8.43 | 125.18 | 177.79 | 52.76 | 18.99 | 8.46 | 125.26 | 177.78 | 52.92 | 19.18 |  |  |  |  |  |  |  |  |  |  |
| 2171 | L | 8.25 | 121.11 | 175.88 | 55.29 | 42.43 | 8.28 | 121.2 |  |  |  |  |  |  |  |  |  |  |  |  |  |
| 2172 | N | 8.45 | 119.46 | 175.21 | 53.23 | 38.63 | 8.49 | 119.57 | 175.19 | 53.42 | 38.85 |  |  |  |  |  |  |  |  |  |  |
| 2173 | I | 8.07 | 120.72 | 176.14 | 61.55 | 38.85 | 8.09 | 120.79 |  |  |  |  |  |  |  |  |  |  |  |  |  |
| 2174 | M |  |  |  |  |  |  |  |  |  |  |  |  |  |  |  |  |  |  |  |  |
| 2175 | N |  |  |  |  |  |  |  |  |  |  |  |  |  |  |  |  |  |  |  |  |
| 2176 | P |  |  |  |  |  |  |  |  |  |  |  |  |  |  |  |  |  |  |  |  |
| 2177 | G |  |  |  |  |  |  |  |  |  |  |  |  |  |  |  |  |  |  |  |  |
| 2178 | H |  |  |  |  |  |  |  |  |  |  |  |  |  |  |  |  |  |  |  |  |
| 2179 | N |  |  |  |  |  |  |  |  |  |  |  |  |  |  |  |  |  |  |  |  |
| 2180 | P |  |  |  |  |  |  |  |  |  |  |  |  |  |  |  |  |  |  |  |  |
| 2181 | N |  |  |  |  |  |  |  |  |  |  |  |  |  |  |  |  |  |  |  |  |
| 2182 | M |  |  | 176.61 |  |  |  |  |  |  |  |  |  |  |  |  |  |  |  |  |  |
| 2183 | A | 8.33 | 124.26 | 178.17 | 53.49 | 18.72 | 8.32 | 124.22 | 178.24 | 53.71 | 18.91 |  |  |  |  |  |  |  |  |  |  |
| 2184 | S | 8.07 | 113.16 |  | 58.73 | 63.54 | 8.07 | 113.09 | 174.72 | 58.93 | 63.73 |  |  |  |  |  |  |  |  |  |  |
| 2185 | M |  |  |  |  |  |  |  |  |  |  |  |  |  |  |  |  |  |  |  |  |
| 2186 | N |  |  |  |  |  |  |  |  |  |  |  |  |  |  |  |  |  |  |  |  |
| 2187 | P |  |  |  |  |  |  |  |  |  |  |  |  |  |  |  |  |  |  |  |  |
| 2188 | Q |  |  |  |  |  |  |  |  |  |  |  |  |  |  |  |  |  |  |  |  |
| 2189 | Y |  |  |  |  |  |  |  |  |  |  |  |  |  |  |  |  |  |  |  |  |
| 2190 | R |  |  |  |  |  |  |  |  |  |  |  |  |  |  |  |  |  |  |  |  |
| 2191 | E |  |  |  |  |  |  |  |  |  |  |  |  |  |  |  |  |  |  |  |  |
| 2192 | M |  |  |  |  |  |  |  |  |  |  |  |  |  |  |  |  |  |  |  |  |
| 2193 | L |  |  |  |  |  |  |  |  |  |  |  |  |  |  |  |  |  |  |  |  |
| 2194 | R |  |  |  |  |  |  |  |  |  |  |  |  |  |  |  |  |  |  |  |  |
| 2195 | R |  |  |  |  |  |  |  |  |  |  |  |  |  |  |  |  |  |  |  |  |
| 2196 | Q |  |  |  |  |  |  |  |  |  |  |  |  |  |  |  |  |  |  |  |  |
| 2197 | L |  |  |  |  |  |  |  |  |  |  |  |  |  |  |  |  |  |  |  |  |
| 2198 | L |  |  |  |  |  |  |  |  |  |  |  |  |  |  |  |  |  |  |  |  |
| 2199 | Q |  |  |  |  |  |  |  |  |  |  |  |  |  |  |  |  |  |  |  |  |
| 2200 | Q |  |  |  |  |  |  |  |  |  |  |  |  |  |  |  |  |  |  |  |  |
| 2201 | Q |  |  |  |  |  |  |  |  |  |  |  |  |  |  |  |  |  |  |  |  |
| 2202 | Q |  |  |  |  |  |  |  | 178.18 | 58.37 | 28.69 |  |  |  |  |  |  |  |  |  |  |
| 2203 | Q |  |  |  |  |  | 8.28 | 119.86 | 177.97 | 58.1 | 28.69 |  |  |  |  |  |  |  |  |  |  |
| 2204 | Q |  |  |  |  |  | 8.26 | 119.94 | 177.86 | 58.26 | 28.7 |  |  |  |  |  |  |  |  |  |  |
| 2205 | Q | 8.27 | 119.98 | 177.67 | 57.83 | 28.64 | 8.26 | 119.97 | 177.72 | 58.07 | 28.67 |  |  |  |  |  |  |  |  |  |  |
| 2206 | Q | 8.28 | 120.11 | 177.55 | 57.61 | 29.46 | 8.28 | 120.11 | 177.62 | 57.85 | 28.94 |  |  |  |  |  |  |  |  |  |  |
| 2207 | Q | 8.3 | 120.18 | 177.46 | 57.45 | 28.75 | 8.31 | 120.19 | 177.5 | 57.76 | 28.94 |  |  |  |  |  |  |  |  |  |  |
| 2208 | Q | 8.32 | 120.25 | 177.37 | 57.34 | 28.81 | 8.32 | 120.27 | 177.38 | 57.5 | 28.99 |  |  |  |  |  |  |  |  |  |  |
| 2209 | Q | 8.33 | 120.33 | 177.2 | 57.28 | 28.92 | 8.33 | 120.36 | 177.24 | 57.49 | 28.99 |  |  |  |  |  |  |  |  |  |  |
| 2210 | Q | 8.34 | 120.4 | 177.11 | 57.17 | 28.86 | 8.35 | 120.44 | 177.11 | 57.25 | 29.11 |  |  |  |  |  |  |  |  |  |  |
| 2211 | Q | 8.35 | 120.56 | 176.84 | 56.95 | 29.03 | 8.37 | 120.54 | 176.97 | 57.16 | 29.16 |  |  |  |  |  |  |  |  |  |  |
| 2212 | Q | 8.38 | 120.63 | 176.72 | 56.74 | 29.03 | 8.38 | 120.67 | 176.83 | 57.04 | 29.27 |  |  |  |  |  |  |  |  |  |  |
| 2213 | Q | 8.4 | 120.82 | 176.67 | 56.63 | 29.14 | 8.4 | 120.81 | 176.67 | 56.79 | 29.29 |  |  |  |  |  |  |  |  |  |  |
| 2214 | Q | 8.43 | 121.05 | 176.59 | 56.63 | 29.19 | 8.43 | 121.03 | 176.49 | 56.54 | 29.38 |  |  |  |  |  |  |  |  |  |  |
| 2215 | Q | 8.47 | 121.35 | 176.07 | 56.35 | 29.25 | 8.48 | 121.31 | 176.37 | 56.43 | 29.46 |  |  |  |  |  |  |  |  |  |  |
| 2216 | Q | 8.54 | 121.73 | 176.8 | 55.8 | 29.25 | 8.56 | 121.72 |  | 56.45 | 29.54 |  |  |  |  |  |  |  |  |  |  |
| 2217 | G | 8.55 | 110.28 | 174.43 | 45.41 |  | 8.57 | 110.49 | 174.42 | 45.69 |  |  |  |  |  |  |  |  |  |  |  |
| 2218 | S | 8.31 | 115.89 | 174.76 | 58.59 | 63.88 | 8.36 | 116 | 174.71 | 58.82 | 64.23 |  |  |  |  |  |  |  |  |  |  |
| 2219 | A | 8.5 | 125.87 | 178.45 | 53.07 | 19.08 | 8.53 | 126.06 | 178.41 | 53.26 | 19.19 |  |  |  |  |  |  |  |  |  |  |
| 2220 | G | 8.39 | 107.76 | 174.43 | 45.37 |  |  |  |  |  |  | 8.44 | 108.11 | 174.37 | 45.55 |  |  |  |  |  |  |
| 2221 | M | 8.19 | 119.92 | 176.4 | 55.49 | 32.86 |  |  |  |  |  | 8.19 | 119.83 | 176.4 | 55.53 | 32.95 |  |  |  |  |  |
| 2222 | A | 8.48 | 125.46 | 178.36 | 52.89 | 19.04 |  |  |  |  |  | 8.46 | 125.24 | 178.36 | 52.92 | 19.09 |  |  |  |  |  |
| 2223 | G | 8.48 | 108.44 | 174.91 | 45.39 |  |  |  |  |  |  | 8.47 | 108.35 | 174.93 | 45.48 |  |  |  |  |  |  |
| 2224 | G | 8.31 | 108.58 | 174.44 | 45.34 |  |  |  |  |  |  | 8.32 | 108.57 | 174.42 | 45.38 |  |  |  |  |  |  |
| 2225 | M | 8.34 | 119.81 | 176.32 | 55.55 |  |  |  |  |  |  | 8.35 | 119.78 | 176.28 | 55.5 | 32.88 |  |  |  |  |  |
| 2226 | A | 8.43 | 124.99 | 178.28 | 52.84 | 19.03 |  |  |  |  |  | 8.44 | 124.93 | 178.29 | 52.84 | 19.13 |  |  |  |  |  |
| 2227 | G | 8.42 | 108.16 |  | 45.32 |  |  |  |  |  |  | 8.42 | 108.15 | 174.19 | 45.34 |  |  |  |  |  |  |
| 2228 | H |  |  | 174.98 |  |  |  |  |  |  |  | 8.39 | 118.28 | 175.45 | 55.75 | 29.61 |  |  |  |  |  |
| 2229 | G | 8.61 | 110.07 | 174.39 | 45.38 |  |  |  |  |  |  | 8.63 | 110.18 | 174.14 | 45.45 |  |  |  |  |  |  |
| 2230 | Q | 8.38 | 119.89 |  | 56 | 29.56 |  |  |  |  |  | 8.37 | 120.02 | 175.78 | 55.99 | 29.44 |  |  |  |  |  |
| 2231 | F | 8.43 | 121.49 | 175.45 | 57.63 | 39.49 |  |  |  |  |  | 8.42 | 121.28 | 175.47 | 57.75 | 39.58 |  |  |  |  |  |
| 2232 | Q | 8.3 | 122.8 | 175.17 | 55.39 | 29.74 |  |  |  |  |  | 8.3 | 122.56 | 175.16 | 55.38 | 29.7 |  |  |  |  |  |
| 2233 | Q | 8.51 | 123.09 |  | 53.84 | 28.8 |  |  |  |  |  | 8.51 | 123.13 |  | 53.81 | 28.78 |  |  |  |  |  |
| 2234 | P |  |  | 176.82 |  |  |  |  |  |  |  |  |  | 176.86 | 63.3 | 32.15 |  |  |  |  |  |
| 2235 | Q | 8.65 | 120.87 | 176.26 | 55.76 | 29.78 |  |  |  |  |  | 8.66 | 120.76 | 176.26 | 55.74 | 29.91 |  |  |  |  |  |
| 2236 | G | 8.34 | 110.4 |  | 44.59 |  |  |  |  |  |  | 8.35 | 110.32 | 172.03 | 44.66 |  |  |  |  |  |  |
| 2237 | P |  |  |  |  |  |  |  |  |  |  |  |  | 177.91 | 63.65 | 32.06 |  |  |  |  |  |
| 2238 | G | 8.63 | 109.6 | 174.5 | 45.28 |  |  |  |  |  |  | 8.65 | 109.58 | 174.53 | 45.3 |  |  |  |  |  |  |
| 2239 | G | 8.17 | 108.21 | 173.53 | 44.93 |  |  |  |  |  |  | 8.19 | 108.18 | 173.53 | 44.97 |  |  |  |  |  |  |
| 2240 | Y | 8.1 | 120.9 |  | 55.85 | 38.16 |  |  |  |  |  | 8.11 | 120.86 | 173.68 | 55.88 | 38.16 |  |  |  |  |  |
| 2241 | P |  |  |  |  |  |  |  |  |  |  |  |  |  |  |  |  |  |  |  |  |
| 2242 | P |  |  | 176.57 |  |  |  |  |  |  |  |  |  | 176.57 | 62.93 | 31.97 |  |  |  |  |  |
| 2243 | A | 8.49 | 124.41 | 177.91 | 52.59 | 19.15 |  |  |  |  |  | 8.5 | 124.31 | 178.03 | 52.61 | 19.21 |  |  |  |  |  |
| 2244 | M | 8.48 | 119.89 |  | 55.66 | 32.79 |  |  |  |  |  | 8.47 | 119.6 | 176.49 | 55.69 | 32.78 |  |  |  |  |  |
| 2245 | Q | 8.52 | 121.51 |  | 56.26 | 29.41 |  |  |  |  |  | 8.5 | 121.58 | 176.29 | 56.25 | 29.33 |  |  |  |  |  |
| 2246 | Q |  |  |  |  |  |  |  |  |  |  |  |  |  |  |  |  |  |  |  |  |
| 2247 | Q |  |  |  |  |  |  |  |  |  |  |  |  |  |  |  |  |  |  |  |  |
| 2248 | Q |  |  |  |  |  |  |  |  |  |  |  |  |  |  |  |  |  |  |  |  |
| 2249 | R |  |  |  |  |  |  |  |  |  |  |  |  |  |  |  |  |  |  |  |  |
| 2250 | M |  |  |  |  |  |  |  |  |  |  |  |  |  |  |  |  |  |  |  |  |
| 2251 | Q |  |  |  |  |  |  |  |  |  |  |  |  | 175.95 |  |  |  |  |  |  |  |
| 2252 | Q | 8.44 | 120.99 |  | 56.61 | 29.05 |  |  |  |  |  | 8.41 | 120.92 | 175.57 | 56 | 29.51 |  |  |  |  |  |
| 2253 | H |  |  |  |  |  |  |  |  |  |  | 8.54 | 119.77 | 174.4 | 55.39 | 29.58 |  |  |  |  |  |
| 2254 | L |  |  |  |  |  |  |  |  |  |  | 8.4 | 124.92 | 175.13 | 53.2 | 41.57 |  |  |  |  |  |
| 2255 | P |  |  | 176.92 | 63.07 |  |  |  |  |  |  |  |  | 176.92 | 62.99 | 32.03 |  |  |  |  |  |
| 2256 | L | 8.49 | 122.54 |  |  |  |  |  |  |  |  | 8.5 | 122.43 | 177.68 | 55.36 | 42.18 |  |  |  |  |  |
| 2257 | Q |  |  | 176.49 | 56.02 |  |  |  |  |  |  | 8.53 | 121.35 | 176.52 | 56.02 | 29.5 |  |  |  |  |  |
| 2258 | G | 8.58 | 110.77 | 174.27 | 45.38 |  |  |  |  |  |  | 8.59 | 110.67 | 174.3 | 45.41 |  |  |  |  |  |  |
| 2259 | S | 8.35 | 115.63 | 174.98 | 58.43 | 64.04 |  |  |  |  |  | 8.37 | 115.63 | 175 | 58.46 | 64.07 |  |  |  |  |  |
| 2260 | S | 8.56 | 118.09 |  | 58.75 | 63.72 |  |  |  |  |  | 8.59 | 118.08 | 174.95 | 58.71 | 63.75 |  |  |  |  |  |
| 2261 | M | 8.49 | 121.92 | 177.15 | 56.16 | 30.66 |  |  |  |  |  | 8.51 | 121.88 | 177.18 | 56.12 | 32.6 |  |  |  |  |  |
| 2262 | G | 8.49 | 109.69 | 174.52 | 45.6 |  |  |  |  |  |  | 8.51 | 109.66 | 174.54 | 45.63 |  |  |  |  |  |  |
| 2263 | Q | 8.3 | 120.18 |  | 56.24 | 29.31 |  |  |  |  |  | 8.32 | 120.16 | 176.63 | 56.36 | 29.3 |  |  |  |  |  |
| 2264 | M |  |  | 176.59 | 55.98 | 32.9 |  |  |  |  |  | 8.47 | 121.2 | 176.59 | 56.01 | 32.68 |  |  |  |  |  |
| 2265 | A | 8.35 | 124.83 | 178.13 | 53.21 | 19.03 |  |  |  |  |  | 8.36 | 124.72 | 178.15 | 53.17 | 19.02 |  |  |  |  |  |
| 2266 | A | 8.28 | 122.61 | 178.23 | 53.07 | 19.02 |  |  |  |  |  | 8.29 | 122.5 | 178.25 | 53.09 | 19.01 |  |  |  |  |  |
| 2267 | Q | 8.3 | 118.94 | 176.46 | 56.06 | 29.31 |  |  |  |  |  | 8.31 | 118.81 | 176.45 | 56.1 | 29.32 |  |  |  |  |  |
| 2268 | M | 8.38 | 120.85 | 177.07 | 55.93 | 32.83 |  |  |  |  |  | 8.4 | 120.77 | 177.1 | 55.9 | 32.71 |  |  |  |  |  |
| 2269 | G | 8.46 | 109.79 |  | 45.51 |  |  |  |  |  |  | 8.48 | 109.72 | 174.31 | 45.53 |  |  |  |  |  |  |
| 2270 | Q | 8.3 | 119.79 | 176.36 | 55.93 | 29.37 |  |  |  |  |  | 8.31 | 119.73 | 176.36 | 55.92 | 29.42 |  |  |  |  |  |
| 2271 | L | 8.41 | 122.88 | 177.17 |  |  |  |  |  |  |  | 8.42 | 122.76 | 178.17 | 55.62 | 42.18 |  |  |  |  |  |
| 2272 | G | 8.49 | 109.48 | 174.27 | 45.58 |  |  |  |  |  |  | 8.5 | 109.41 | 174.29 | 45.47 | 45.47 |  |  |  |  |  |
| 2273 | Q | 8.3 | 119.77 | 176.26 | 55.9 | 29.34 |  |  |  |  |  | 8.3 | 119.68 | 176.27 | 55.9 | 29.45 |  |  |  |  |  |
| 2274 | M | 8.54 | 121.08 |  | 55.79 |  |  |  |  |  |  | 8.55 | 121.19 | 176.84 | 55.73 | 32.65 |  |  |  |  |  |
| 2275 | G | 8.5 | 110.14 | 173.69 | 45.16 |  |  |  |  |  |  | 8.52 | 110.06 | 173.72 | 45.21 |  |  |  |  |  |  |
| 2276 | Q | 8.28 | 120.71 |  | 53.76 | 28.87 |  |  |  |  |  | 8.29 | 120.64 | 174.14 | 53.64 | 29.08 |  |  |  |  |  |
| 2277 | P |  |  | 177.6 | 63.65 |  |  |  |  |  |  |  |  | 177.53 | 63.5 | 32.15 |  |  |  |  |  |
| 2278 | G | 8.61 | 109.37 | 174.16 | 45.22 |  |  |  |  |  |  | 8.61 | 109.32 | 174.21 | 45.27 |  |  |  |  |  |  |
| 2279 | L | 8.23 | 121.71 | 178.07 | 55.31 | 42.44 |  |  |  |  |  | 8.24 | 121.65 | 178.13 | 55.23 | 42.48 |  |  |  |  |  |
| 2280 | G | 8.6 | 110.26 | 174.27 | 45.26 |  |  |  |  |  |  | 8.62 | 110.19 | 174.3 | 45.27 |  |  |  |  |  |  |
| 2281 | A | 8.33 | 124.07 | 177.73 | 52.88 | 19.22 |  |  |  |  |  | 8.35 | 124.04 | 177.75 | 52.88 | 19.34 |  |  |  |  |  |
| 2282 | D | 8.49 | 118.79 | 176.12 | 54.05 | 40.13 |  |  |  |  |  | 8.48 | 118.91 | 176.33 | 54.32 | 41.01 |  |  |  |  |  |
| 2283 | S | 8.24 | 116.17 | 174.41 | 58.49 | 63.89 |  |  |  |  |  | 8.25 | 116.07 | 174.43 | 58.4 | 63.88 |  |  |  |  |  |
| 2284 | T | 8.27 | 118.26 | 173.13 | 60.57 | 69.58 |  |  |  |  |  | 8.29 | 118.32 | 173.04 | 60.59 | 69.51 |  |  |  |  |  |
| 2285 | P |  |  | 176.97 |  |  |  |  |  |  |  |  |  |  | 63.75 | 32.12 |  |  |  |  |  |
| 2286 | N | 8.56 | 119.13 | 176.06 | 53.63 | 38.51 |  |  |  |  |  | 8.58 | 119.1 | 176.05 | 53.69 | 38.52 |  |  |  |  |  |
| 2287 | I | 8.2 | 121.96 | 177.16 |  |  |  |  |  |  |  | 8.21 | 121.86 | 177.14 | 62.65 | 38.39 |  |  |  |  |  |
| 2288 | Q | 8.41 | 122.27 |  | 57.65 | 28.6 |  |  |  |  |  | 8.43 | 122.25 | 177.55 | 57.66 | 28.57 |  |  |  |  |  |
| 2289 | Q | 8.4 | 120.89 | 177.34 | 56.75 | 29.08 |  |  |  |  |  | 8.41 | 120.89 | 177.33 | 57.67 | 29.58 |  |  |  |  |  |
| 2290 | A | 8.28 | 123.54 | 179.61 | 54.15 | 18.54 |  |  |  |  |  | 8.3 | 123.52 | 179.6 | 54.11 | 18.52 |  |  |  |  |  |
| 2291 | L | 8.14 | 120.6 |  |  |  |  |  |  |  |  | 8.15 | 120.55 | 178.53 | 57.05 | 41.81 |  |  |  |  |  |
| 2292 | Q | 8.2 | 119.05 | 177.83 | 57.69 | 28.44 |  |  |  |  |  | 8.21 | 118.97 | 177.82 | 57.77 | 28.53 |  |  |  |  |  |
| 2293 | Q | 8.25 | 119.46 |  | 57.75 | 28.5 |  |  |  |  |  | 8.26 | 119.41 | 177.41 | 57.79 | 28.61 |  |  |  |  |  |
| 2294 | R | 8.08 | 121.12 | 177.99 | 58.29 |  |  |  |  |  |  |  |  |  |  |  | 8.31 | 121.36 | 178.28 | 58.61 | 30.25 |
| 2295 | I | 8.13 | 121.36 | 177.96 |  |  |  |  |  |  |  |  |  |  |  |  | 8.25 | 121.37 | 178.17 | 63.8 | 38.18 |
| 2296 | L | 8.11 | 123.03 |  |  |  |  |  |  |  |  |  |  |  |  |  | 7.99 | 122.41 | 178.99 | 57.12 | 41.83 |
| 2297 | Q |  |  |  |  |  |  |  |  |  |  |  |  |  |  |  | 8.21 | 119.44 | 177.99 | 57.99 | 28.84 |
| 2298 | Q | 8.05 | 119.21 |  |  |  |  |  |  |  |  |  |  |  |  |  | 8.07 | 119.45 | 177.83 | 57.78 | 28.62 |
| 2299 | Q | 8.27 | 119.81 |  | 57.82 | 28.66 |  |  |  |  |  |  |  |  |  |  | 8.28 | 119.69 | 177.52 | 28.85 | 57.53 |
| 2300 | Q | 8.29 | 119.79 | 177.01 | 57.51 | 28.78 |  |  |  |  |  |  |  |  |  |  | 8.27 | 119.55 | 177.18 | 57.07 | 28.9 |
| 2301 | M | 8.2 | 120.29 | 176.86 |  |  |  |  |  |  |  |  |  |  |  |  | 8.18 | 119.93 | 177 | 56.51 | 32.66 |
| 2302 | K | 8.19 | 121.62 | 176.89 | 56.96 | 32.78 |  |  |  |  |  |  |  |  |  |  | 8.16 | 121.22 | 177.07 | 57.08 | 32.81 |
| 2303 | Q | 8.3 | 120.78 | 176.03 | 56.13 | 29.34 |  |  |  |  |  |  |  |  |  |  | 8.27 | 120.34 | 176.1 | 56.16 | 29.38 |
| 2304 | Q | 8.43 | 121.79 | 176.04 | 55.69 | 29.47 |  |  |  |  |  |  |  |  |  |  | 8.4 | 121.43 | 176.12 | 55.92 | 29.37 |
| 2305 | I | 8.28 | 121.66 | 176.8 | 61.47 | 38.78 |  |  |  |  |  |  |  |  |  |  | 8.31 | 122.4 | 176.89 | 61.6 | 38.65 |
| 2306 | G | 8.57 | 113.21 | 173.79 | 45.04 |  |  |  |  |  |  |  |  |  |  |  | 8.58 | 113.04 | 173.81 | 45.08 |  |
| 2307 | S | 8.29 | 117.09 | 172.8 | 56.57 | 63.42 |  |  |  |  |  |  |  |  |  |  | 8.31 | 117.04 | 172.84 | 56.53 | 63.38 |
| 2308 | P |  |  | 177.68 |  |  |  |  |  |  |  |  |  |  |  |  |  |  | 177.68 | 63.77 | 32.07 |
| 2309 | G | 8.58 | 109.5 | 173.87 | 45.04 |  |  |  |  |  |  |  |  |  |  |  | 8.61 | 109.49 | 173.88 | 45.15 |  |
| 2310 | Q | 8.18 | 120.67 |  | 53.53 | 28.94 |  |  |  |  |  |  |  |  |  |  | 8.19 | 120.6 | 173.97 | 53.45 | 29.06 |
| 2311 | P |  |  | 176.38 |  | 63.22 |  |  |  |  |  |  |  |  |  |  |  |  |  | 63.11 | 32.18 |
| 2312 | N | 8.7 | 120.29 |  | 51.26 | 38.7 |  |  |  |  |  |  |  |  |  |  | 8.71 | 120.25 | 173.8 | 51.25 | 38.82 |
| 2313 | P |  |  | 176.97 | 63.54 | 32.25 |  |  |  |  |  |  |  |  |  |  |  |  | 176.99 | 63.55 | 32.26 |
| 2314 | M | 8.42 | 119.21 | 176.28 | 55.16 | 32.59 |  |  |  |  |  |  |  |  |  |  | 8.44 | 119.17 | 176.29 | 55.26 | 32.84 |
| 2315 | S | 8.28 | 118.53 | 173.01 | 56.67 | 63.23 |  |  |  |  |  |  |  |  |  |  | 8.3 | 118.37 | 172.92 | 56.62 | 63.24 |
| 2316 | P |  |  |  |  |  |  |  |  |  |  |  |  |  |  |  |  |  | 177.36 |  |  |
| 2317 | Q |  |  |  |  |  |  |  |  |  |  |  |  |  |  |  | 8.52 | 119.81 | 176.39 | 56.32 | 29.31 |
| 2318 | Q |  |  |  |  |  |  |  |  |  |  |  |  |  |  |  | 8.39 | 121.21 | 176.16 | 56.34 | 29.42 |
| 2319 | H |  |  |  |  |  |  |  |  |  |  |  |  |  |  |  | 8.53 | 119.9 | 175.28 | 56.2 | 29.33 |
| 2320 | M |  |  | 176.4 |  |  |  |  |  |  |  |  |  |  |  |  | 8.4 | 121.39 | 176.43 | 55.73 | 32.8 |
| 2321 | L | 8.14 | 122.1 | 177.57 | 55.05 | 42.15 |  |  |  |  |  |  |  |  |  |  | 8.42 | 123.25 | 177.62 | 55.35 | 42.29 |
| 2322 | S | 8.39 | 116.7 | 175.07 | 58.61 | 63.97 |  |  |  |  |  |  |  |  |  |  | 8.38 | 116.43 | 175.15 | 58.67 | 63.81 |
| 2323 | G | 8.5 | 110.76 | 173.9 | 45.23 |  |  |  |  |  |  |  |  |  |  |  | 8.51 | 110.73 | 173.92 | 45.25 |  |
| 2324 | Q | 8.27 | 120.69 |  | 53.57 | 28.89 |  |  |  |  |  |  |  |  |  |  | 8.25 | 120.57 | 174.16 | 53.68 | 28.88 |
| 2325 | P |  |  | 177.01 | 63.2 |  |  |  |  |  |  |  |  |  |  |  |  |  | 177 | 63.28 | 32.11 |
| 2326 | Q | 8.65 | 121.12 |  | 55.68 | 29.63 |  |  |  |  |  |  |  |  |  |  | 8.66 | 121.04 | 175.91 | 55.72 | 29.59 |
| 2327 | A | 8.49 | 125.85 | 177.63 | 52.59 | 19.15 |  |  |  |  |  |  |  |  |  |  | 8.51 | 125.81 | 177.75 | 52.65 | 19.37 |
| 2328 | S | 8.38 | 115.18 | 174.91 | 58.33 | 63.94 |  |  |  |  |  |  |  |  |  |  | 8.42 | 115.07 | 174.21 | 58.43 | 63.86 |
| 2329 | H | 8.29 | 121.7 | 180.79 | 57.01 | 30.03 |  |  |  |  |  |  |  |  |  |  | 8.46 | 120.66 | 174.54 | 55.55 | 30.09 |
| 2330 | L | 7.9 | 121.4 | 177.55 | 55.3 | 42.27 |  |  |  |  |  |  |  |  |  |  | 8.35 | 124.89 | 175.18 | 53.14 | 41.49 |
| 2331 | P |  |  | 177.74 | 63.47 |  |  |  |  |  |  |  |  |  |  |  |  |  | 177.84 | 63.59 | 32.04 |
| 2332 | G | 8.65 | 109.7 |  | 45.38 |  |  |  |  |  |  |  |  |  |  |  | 8.7 | 109.79 | 174.42 | 45.44 |  |
| 2333 | Q | 8.19 | 119.77 |  | 55.65 | 29.41 |  |  |  |  |  |  |  |  |  |  | 8.21 | 119.68 | 176.04 | 55.86 | 29.54 |
| 2334 | Q | 8.6 | 122.1 | 175.91 | 55.82 | 29.28 |  |  |  |  |  |  |  |  |  |  | 8.59 | 122.12 | 175.96 | 55.83 | 29.4 |
| 2335 | I | 8.34 | 122.98 | 176.12 | 61.14 | 38.81 |  |  |  |  |  |  |  |  |  |  | 8.34 | 122.85 | 176.15 | 61.13 | 38.8 |
| 2336 | A | 8.57 | 128.83 | 177.98 | 52.69 | 19.23 |  |  |  |  |  |  |  |  |  |  | 8.58 | 128.63 | 178.04 | 52.76 | 19.31 |
| 2337 | T | 8.26 | 114.12 |  | 62.01 | 69.85 |  |  |  |  |  |  |  |  |  |  | 8.28 | 114 | 174.82 | 62.07 | 69.87 |
| 2338 | S | 8.41 | 118.22 |  | 58.53 | 63.82 |  |  |  |  |  |  |  |  |  |  | 8.43 | 118.16 | 174.85 | 58.46 | 63.77 |
| 2339 | L | 8.42 | 124.31 |  |  |  |  |  |  |  |  |  |  |  |  |  | 8.43 | 124.22 | 177.89 | 55.54 | 42.23 |
| 2340 | S |  |  | 174.56 | 58.84 | 63.76 |  |  |  |  |  |  |  |  |  |  | 8.35 | 115.94 | 174.61 | 58.75 | 63.7 |
| 2341 | N | 8.44 | 120.55 | 175.18 | 53.36 | 38.62 |  |  |  |  |  |  |  |  |  |  | 8.46 | 120.51 | 175.2 | 53.42 | 38.7 |
| 2342 | Q | 8.28 | 120.4 | 175.96 | 55.96 | 29.53 |  |  |  |  |  |  |  |  |  |  | 8.29 | 120.3 | 175.97 | 55.98 | 29.57 |
| 2343 | V | 8.28 | 122.22 | 176.14 | 62.67 | 29.56 |  |  |  |  |  |  |  |  |  |  | 8.28 | 122.08 | 176.16 | 62.54 | 32.64 |
| 2344 | R | 8.55 | 125.83 | 176.05 | 55.69 | 30.98 |  |  |  |  |  |  |  |  |  |  | 8.56 | 125.7 | 176.09 | 55.78 | 31.02 |
| 2345 | S | 8.53 | 119.51 |  | 56.52 | 63.13 |  |  |  |  |  |  |  |  |  |  | 8.56 | 119.4 | 172.45 | 56.56 | 63.18 |
| 2346 | P |  |  | 176.31 |  |  |  |  |  |  |  |  |  |  |  |  |  |  | 176.31 | 62.97 | 32.1 |
| 2347 | A | 8.46 | 126.29 |  | 50.53 | 17.91 |  |  |  |  |  |  |  |  |  |  | 8.48 | 126.23 | 175.6 | 50.53 | 18.01 |
| 2348 | P |  |  | 176.94 | 62.85 | 32.12 |  |  |  |  |  |  |  |  |  |  |  |  | 176.97 | 62.87 | 32.13 |
| 2349 | V | 8.39 | 121.34 | 176.34 | 56.09 | 29.52 |  |  |  |  |  |  |  |  |  |  | 8.4 | 121.2 | 176.35 | 62.39 | 32.9 |
| 2350 | Q | 8.62 | 125.09 | 175.69 | 55.44 | 29.65 |  |  |  |  |  |  |  |  |  |  | 8.63 | 124.93 | 175.71 | 55.44 | 29.78 |
| 2351 | S | 8.58 | 119.95 |  | 56.74 | 63.11 |  |  |  |  |  |  |  |  |  |  | 8.6 | 119.85 | 172.54 | 56.68 | 63.13 |
| 2352 | P |  |  |  |  |  |  |  |  |  |  |  |  |  |  |  |  |  |  |  |  |
| 2353 | R |  |  |  |  |  |  |  |  |  |  |  |  |  |  |  |  |  |  |  |  |
| 2354 | P |  |  | 177.01 |  |  |  |  |  |  |  |  |  |  |  |  |  |  | 177.04 | 63.2 | 32.15 |
| 2355 | Q | 8.69 | 121.21 |  | 55.96 | 29.52 |  |  |  |  |  |  |  |  |  |  | 8.71 | 121.11 | 176.19 | 55.95 | 29.6 |
| 2356 | S | 8.48 | 117.74 | 174.09 | 58.37 | 63.89 |  |  |  |  |  |  |  |  |  |  | 8.49 | 117.53 | 174.12 | 58.37 | 63.86 |
| 2357 | Q | 8.51 | 123.04 |  | 53.69 | 29 |  |  |  |  |  |  |  |  |  |  | 8.51 | 123.02 | 173.56 | 53.59 | 29.13 |
| 2358 | P |  |  |  |  |  |  |  |  |  |  |  |  |  |  |  |  |  |  |  |  |
| 2359 | P |  |  |  |  |  |  |  |  |  |  |  |  |  |  |  |  |  |  | 62.98 | 32.12 |
| 2360 | H |  |  |  |  |  |  |  |  |  |  |  |  |  |  |  | 8.62 | 119.08 | 175.02 | 55.88 | 29.83 |
| 2361 | S | 8.47 | 117.73 | 174.09 | 58.17 | 63.96 |  |  |  |  |  |  |  |  |  |  | 8.47 | 117.58 | 174.12 | 58.24 | 64.02 |
| 2362 | S | 8.58 | 119.37 |  | 56.68 | 63.24 |  |  |  |  |  |  |  |  |  |  | 8.56 | 119.33 | 172.48 | 56.6 | 63.34 |
| 2363 | P |  |  |  |  |  |  |  |  |  |  |  |  |  |  |  |  |  |  |  |  |
| 2364 | S |  |  |  |  |  |  |  |  |  |  |  |  |  |  |  |  |  |  |  |  |
| 2365 | P |  |  | 176.67 |  |  |  |  |  |  |  |  |  |  |  |  |  |  | 176.72 | 63.18 | 32.21 |
| 2366 | R | 8.49 | 121.96 | 176.27 | 56.37 | 30.67 |  |  |  |  |  |  |  |  |  |  | 8.51 | 121.92 | 176.34 | 56.22 | 30.81 |
| 2367 | I | 8.34 | 123.53 | 176.06 | 60.75 | 38.86 |  |  |  |  |  |  |  |  |  |  | 8.34 | 123.3 | 176.08 | 60.75 | 38.61 |
| 2368 | Q | 8.65 | 126.98 | 173.88 | 53.48 | 29 |  |  |  |  |  |  |  |  |  |  | 8.65 | 126.75 | 173.83 | 53.51 | 28.99 |
| 2369 | P |  |  |  |  |  |  |  |  |  |  |  |  |  |  |  |  |  |  |  |  |
| 2370 | Q |  |  |  |  |  |  |  |  |  |  |  |  |  |  |  |  |  |  |  |  |
| 2371 | P |  |  |  |  |  |  |  |  |  |  |  |  |  |  |  |  |  |  |  |  |
| 2372 | S |  |  |  |  |  |  |  |  |  |  |  |  |  |  |  |  |  |  |  |  |
| 2373 | P |  |  |  |  |  |  |  |  |  |  |  |  |  |  |  |  |  |  | 63.33 | 32.11 |
| 2374 | H |  |  |  |  |  |  |  |  |  |  |  |  |  |  |  | 8.46 | 118.98 | 174.71 | 55.72 | 29.99 |
| 2375 | H |  |  |  | 55.76 | 29.39 |  |  |  |  |  |  |  |  |  |  | 8.51 | 121.13 | 174.6 | 56.31 | 30.15 |
| 2376 | V | 8.32 | 122.64 | 175.84 |  |  |  |  |  |  |  |  |  |  |  |  | 8.34 | 122.71 | 175.86 | 61.97 | 33.09 |
| 2377 | S | 8.67 | 122.33 |  | 56.65 | 63.22 |  |  |  |  |  |  |  |  |  |  | 8.65 | 122.02 | 172.8 | 56.52 | 63.29 |
| 2378 | P |  |  | 176.94 | 63.28 |  |  |  |  |  |  |  |  |  |  |  |  |  |  | 63.33 | 32.17 |
| 2379 | Q | 8.62 | 121 |  | 55.89 | 29.37 |  |  |  |  |  |  |  |  |  |  | 8.63 | 120.8 | 176.44 | 55.93 | 29.44 |
| 2380 | T | 8.32 | 115.78 | 175.08 | 62.02 | 69.81 |  |  |  |  |  |  |  |  |  |  | 8.32 | 115.49 | 175.14 | 62.12 | 69.86 |
| 2381 | G | 8.54 | 111.48 | 173.81 | 45.1 |  |  |  |  |  |  |  |  |  |  |  | 8.56 | 111.53 | 173.86 | 45.13 |  |
| 2382 | S | 8.32 | 117.07 |  | 56.54 | 63.41 |  |  |  |  |  |  |  |  |  |  | 8.34 | 117.12 | 172.71 | 56.49 | 63.4 |
| 2383 | P |  |  |  |  |  |  |  |  |  |  |  |  |  |  |  |  |  |  |  |  |
| 2384 | H |  |  |  |  |  |  |  |  |  |  |  |  |  |  |  |  |  |  |  |  |
| 2385 | P |  |  | 177.54 | 63.73 |  |  |  |  |  |  |  |  |  |  |  |  |  |  | 63.73 | 32.15 |
| 2386 | G | 8.67 | 109.92 | 173.97 | 45.06 |  |  |  |  |  |  |  |  |  |  |  | 8.85 | 109.92 | 174.13 | 45.21 |  |
| 2387 | L | 8.15 | 122 | 177.24 | 55.3 | 42.69 |  |  |  |  |  |  |  |  |  |  | 8.13 | 121.86 | 177.27 | 55.14 | 42.61 |
| 2388 | A | 8.15 | 125.4 | 177.88 | 52.69 | 19.23 |  |  |  |  |  |  |  |  |  |  | 8.43 | 125.37 | 177.91 | 52.63 | 19.11 |
| 2389 | V | 8.33 | 119.92 | 176.53 | 62.36 | 33.01 |  |  |  |  |  |  |  |  |  |  | 8.22 | 119.76 | 176.59 | 62.48 | 32.83 |
| 2390 | T | 8.32 | 118.64 | 174.57 | 62.01 | 69.91 |  |  |  |  |  |  |  |  |  |  | 8.33 | 118.44 | 174.62 | 62.08 | 69.88 |
| 2391 | M | 8.54 | 123.6 | 176.09 | 55.48 |  |  |  |  |  |  |  |  |  |  |  | 8.54 | 123.45 | 176.13 | 55.46 | 33 |
| 2392 | A | 8.46 | 125.77 | 177.92 | 52.89 | 19.18 |  |  |  |  |  |  |  |  |  |  | 8.46 | 125.66 | 177.92 | 52.89 | 19.23 |
| 2393 | S | 8.4 | 115.07 | 174.78 | 58.44 | 63.73 |  |  |  |  |  |  |  |  |  |  | 8.42 | 115.12 | 174.83 | 58.45 | 63.89 |
| 2394 | S | 8.4 | 117.88 | 174.79 | 58.62 | 63.67 |  |  |  |  |  |  |  |  |  |  | 8.43 | 118.04 | 174.79 | 58.56 | 63.8 |
| 2395 | I | 8.16 | 122.1 | 176.16 | 61.56 | 38.97 |  |  |  |  |  |  |  |  |  |  | 8.18 | 122.06 | 176.16 | 61.51 | 38.81 |
| 2396 | D | 8.42 | 123.94 | 176.37 | 54.24 | 40.52 |  |  |  |  |  |  |  |  |  |  | 8.43 | 124.05 | 176.51 | 54.47 | 41.19 |
| 2397 | Q | 8.48 | 121.51 | 176.82 | 56.43 | 29.22 |  |  |  |  |  |  |  |  |  |  | 8.5 | 121.52 | 176.87 | 56.31 | 29.21 |
| 2398 | G | 8.51 | 108.81 | 174.34 | 45.46 |  |  |  |  |  |  |  |  |  |  |  | 8.56 | 109.09 | 174.24 | 45.58 |  |
| 2399 | H |  |  |  |  |  |  |  |  |  |  |  |  |  |  |  | 8.33 | 118.64 | 175.14 | 55.94 | 29.75 |
| 2400 | L |  |  | 177.78 | 55.57 | 42.37 |  |  |  |  |  |  |  |  |  |  | 8.3 | 123.82 | 177.85 | 55.41 | 42.23 |
| 2401 | G | 8.36 | 109.43 | 173.48 | 44.94 |  |  |  |  |  |  |  |  |  |  |  | 8.46 | 109.28 | 173.51 | 45.06 |  |
| 2402 | N | 8.4 | 119.45 |  | 51.58 | 38.74 |  |  |  |  |  |  |  |  |  |  | 8.41 | 119.42 | 173.75 | 51.41 | 38.75 |
| 2403 | P |  |  | 177.39 | 63.86 | 32.09 |  |  |  |  |  |  |  |  |  |  |  |  | 177.35 | 63.79 | 32.13 |
| 2404 | E | 8.54 | 120.13 | 177 | 57.12 | 29.92 |  |  |  |  |  |  |  |  |  |  | 8.59 | 120.24 | 176.97 | 57.04 | 29.89 |
| 2405 | Q | 8.35 | 121.17 | 176.31 | 56.24 | 29.33 |  |  |  |  |  |  |  |  |  |  | 8.35 | 121.02 | 176.37 | 56.18 | 29.43 |
| 2406 | S | 8.39 | 117.02 | 177 | 58.73 | 63.79 |  |  |  |  |  |  |  |  |  |  | 8.4 | 116.99 | 174.51 | 58.79 | 63.71 |
| 2407 | A | 8.35 | 125.69 | 177.56 | 52.5 | 19.2 |  |  |  |  |  |  |  |  |  |  | 8.37 | 125.67 | 177.61 | 52.65 | 19.23 |
| 2408 | M | 8.26 | 119.11 | 176.01 | 55.24 | 32.9 |  |  |  |  |  |  |  |  |  |  | 8.27 | 119.08 | 176.07 | 55.29 | 32.96 |
| 2409 | L | 8.24 | 125.01 | 183.32 | 53.38 | 41.36 |  |  |  |  |  |  |  |  |  |  | 8.26 | 124.92 | 175.24 | 53.23 | 41.51 |
| 2410 | P |  |  | 176.88 | 63.8 | 32.82 |  |  |  |  |  |  |  |  |  |  |  |  | 176.9 | 63.25 | 32.08 |
| 2411 | Q | 8.59 | 120.85 | 175.99 | 55.61 | 29.5 |  |  |  |  |  |  |  |  |  |  | 8.61 | 120.78 | 176.03 | 55.6 | 29.5 |
| 2412 | L | 8.41 | 123.85 |  |  |  |  |  |  |  |  |  |  |  |  |  | 8.43 | 123.81 | 177.13 | 55.19 | 42.45 |
| 2413 | N | 8.57 | 119.59 | 174.86 | 53.22 | 38.89 |  |  |  |  |  |  |  |  |  |  | 8.6 | 119.61 | 174.91 | 53.16 | 39.02 |
| 2414 | T | 8.17 | 117 | 175.5 | 59.69 | 69.73 |  |  |  |  |  |  |  |  |  |  | 8.2 | 117.01 | 172.86 | 59.97 | 69.75 |
| 2415 | P |  |  | 177.08 |  |  |  |  |  |  |  |  |  |  |  |  |  |  | 177.1 | 63.36 | 32.23 |
| 2416 | S | 8.53 | 116.51 |  | 58.51 | 63.77 |  |  |  |  |  |  |  |  |  |  | 8.57 | 116.6 | 174.91 | 58.49 | 63.73 |
| 2417 | R | 8.56 | 123.56 | 176.59 |  |  |  |  |  |  |  |  |  |  |  |  | 8.58 | 123.5 | 176.64 | 56.42 | 30.76 |
| 2418 | S | 8.43 | 117.14 | 174.64 | 58.52 | 63.75 |  |  |  |  |  |  |  |  |  |  | 8.47 | 117.18 | 174.56 | 58.55 | 63.78 |
| 2419 | A | 8.45 | 126.3 | 177.77 | 52.02 | 20.56 |  |  |  |  |  |  |  |  |  |  | 8.46 | 126.24 | 177.97 | 52.81 | 19.19 |
| 2420 | L |  |  |  |  |  |  |  |  |  |  |  |  |  |  |  | 8.28 | 120.87 | 177.85 | 55.28 | 42.37 |
| 2421 | S |  |  |  |  |  |  |  |  |  |  |  |  |  |  |  | 8.35 | 116.51 | 175.1 | 58.71 | 63.71 |
| 2422 | S | 8.41 | 118.07 | 174.86 | 58.35 | 63.87 |  |  |  |  |  |  |  |  |  |  | 8.45 | 118.02 | 174.9 | 58.82 | 63.67 |
| 2423 | E | 8.53 | 122.47 |  |  |  |  |  |  |  |  |  |  |  |  |  | 8.49 | 122.59 | 176.81 | 57 | 29.97 |
| 2424 | L | 8.21 | 122.39 | 177.57 | 55.76 | 42.47 |  |  |  |  |  |  |  |  |  |  | 8.22 | 122.35 | 177.6 | 55.41 | 42.3 |
| 2425 | S | 8.29 | 116.44 | 174.59 | 58.37 | 63.71 |  |  |  |  |  |  |  |  |  |  | 8.32 | 116.53 | 174.61 | 58.46 | 63.67 |
| 2426 | L | 8.1 | 120.37 | 181.52 | 55.37 | 42.3 |  |  |  |  |  |  |  |  |  |  | 8.37 | 124.41 | 177.59 | 55.25 | 42.23 |
| 2427 | V | 8.1 | 120.4 | 176.69 | 62.41 | 32.69 |  |  |  |  |  |  |  |  |  |  | 8.13 | 120.42 | 176.73 | 62.63 | 32.7 |
| 2428 | G | 8.47 | 112.5 | 176.36 | 45.35 |  |  |  |  |  |  |  |  |  |  |  | 8.5 | 112.52 | 173.73 | 45.19 |  |
| 2429 | D | 8.34 | 121.21 | 176.79 | 54.39 | 41.39 |  |  |  |  |  |  |  |  |  |  | 8.37 | 120.8 | 176.99 | 54.32 | 41.41 |
| 2430 | T | 8.41 | 114.82 | 175.27 | 61.87 | 69.46 |  |  |  |  |  |  |  |  |  |  | 8.43 | 114.8 | 175.28 | 61.77 | 69.66 |
| 2431 | T | 8.36 | 116.31 | 175.13 | 60.62 | 68 |  |  |  |  |  |  |  |  |  |  | 8.39 | 116.08 | 175.49 | 62.72 | 69.84 |
| 2432 | G | 8.53 | 111.17 | 173.65 | 45.14 |  |  |  |  |  |  |  |  |  |  |  | 8.55 | 111.22 | 174.09 | 45.41 |  |
| 2433 | D | 8.14 | 121.36 | 176.68 | 54.58 | 41.43 |  |  |  |  |  |  |  |  |  |  | 8.36 | 120.85 | 176.75 | 54.53 | 41.28 |
| 2434 | T | 8.24 | 114.24 | 174.77 | 62.02 | 69.84 |  |  |  |  |  |  |  |  |  |  | 8.26 | 114.61 | 174.71 | 62.1 | 69.75 |
| 2435 | L | 8.4 | 124.5 | 176.96 | 55.68 | 42.31 |  |  |  |  |  |  |  |  |  |  | 8.37 | 124.53 | 177.52 | 55.42 | 42.23 |
| 2436 | E | 8.23 | 122.37 | 176.26 | 56.87 | 30.29 |  |  |  |  |  |  |  |  |  |  | 8.44 | 122.11 | 176.24 | 56.75 | 30.17 |
| 2437 | K | 8.36 | 122.53 | 176.16 | 56.19 | 33.46 |  |  |  |  |  |  |  |  |  |  | 8.3 | 122.4 | 176.15 | 56.12 | 33.18 |
| 2438 | F | 8.43 | 122.45 | 175.58 | 57.9 | 39.56 |  |  |  |  |  |  |  |  |  |  | 8.43 | 122.55 | 175.51 | 57.93 | 39.61 |
| 2439 | V | 8.12 | 123.37 | 175.61 | 61.98 | 33.13 |  |  |  |  |  |  |  |  |  |  | 8.1 | 123.89 | 175.51 | 62.05 | 33.17 |
| 2440 | E | 8.45 | 125.45 | 176.88 | 57.05 | 30.39 |  |  |  |  |  |  |  |  |  |  | 8.49 | 125.38 | 176.87 | 56.9 | 30.38 |
| 2441 | G | 8.47 | 110.5 | 173.35 | 45.58 |  |  |  |  |  |  |  |  |  |  |  | 8.53 | 110.91 | 173.35 | 45.42 |  |
| 2442 | L | 7.85 | 127.06 | 174.71 | 56.64 | 43.2 |  |  |  |  |  |  |  |  |  |  | 7.86 | 127.32 | 172.18 | 56.59 | 43.21 |

**Table S3** *Experimental parameters used for the acquisition of the NMR experiments on full-length ID5 for sequence-specific assignment*

|  | Spectral widths and maximal evolution times | | | | No. of scans | Inter-scan delays (s) | No. of complex points (aq) | No. of hypercomplex points | Duration of the experiment | Relative data points density (%) |
| --- | --- | --- | --- | --- | --- | --- | --- | --- | --- | --- |
| 2D BEST-TROSY | 2300 Hz (^15^N) 112.5 ms | | 10200 Hz (^1^H^N^) 403.0 ms | | 16 | 0.100 | 4096 | 256 | 1 h  10 min | 100.0 |
| 3D BT-HNCO | 2400 Hz (^13^C')  30.8 ms | 2400 Hz (^15^N)  30.8 ms | | 10800 Hz (^1^H^N^) 95.1 ms | 16 | 0.200 | 1024 | 2000 | 15 h 20 min | 35.5 |
| 3D BT-HN(CA)CO | 2400 Hz (^13^C')  30.8 ms | 2400 Hz (^15^N)  30.8 ms | | 10800 Hz (^1^H^N^) 95.1 ms | 32 | 0.200 | 1024 | 2000 | 1 d  6 h  50 min | 35.5 |
| 3D BT-HNCACB | 15000 Hz (^13^C^α/β^) 14.0 ms | 2400 Hz (^15^N)  30.8 ms | | 10800 Hz (^1^H^N^) 95.1 ms | 32 | 0.200 | 1024 | 3600 | 1 d  5 h  50 min | 22.7 |
| 3D BT-HN(CO)CACB | 15000 Hz (^13^C^α/β^) 14.0 ms | 2400 Hz (^15^N)  30.8 ms | | 10800 Hz (^1^H^N^) 95.1 ms | 16 | 0.200 | 1024 | 3600 | 2 d  8 h | 22.7 |

**Table S4** *Experimental parameters used for the acquisition of the NMR experiments on ID5_F1 for sequence-specific assignment*

|  | Spectral widths and maximal evolution times | | | | No. of scans | Inter-scan delays (s) | No. of complex points (aq) | No. of hypercomplex points | Duration of the experiment | Relative data points density (%) |
| --- | --- | --- | --- | --- | --- | --- | --- | --- | --- | --- |
| 2D BEST-TROSY | 2900 Hz (^15^N) 88.6 ms | | 11400 Hz (^1^H^N^) 179.2 ms | | 8 | 0.100 | 2048 | 256 | 25 min | 100.0 |
| 3D BT-HNCO | 1800 Hz (^13^C')  41.1 ms | 2200 Hz (^15^N)  40.5 ms | | 13300 Hz (^1^H^N^) 154.1 ms | 16 | 0.150 | 2048 | 1688 | 12 h 50 min | 25.0 |
| 3D BT-HN(CA)CO | 1800 Hz (^13^C')  41.1 ms | 2200 Hz (^15^N)  40.5 ms | | 14300 Hz (^1^H^N^) 143.8 ms | 32 | 0.150 | 2048 | 1688 | 1 d 2 h  20 min | 25.0 |
| 3D BT-HNCACB | 15700 Hz (^13^C')  20.0 ms | 2200 Hz (^15^N)  40.5 ms | | 14300 Hz (^1^H^N^) 143.8 ms | 16 | 0.150 | 2048 | 5100 | 1 d 15 h  20 min | 18.0 |
| 3D BT-HN(CO)CACB | 15700 Hz (^13^C')  20.0 ms | 2200 Hz (^15^N)  40.5 ms | | 14300 Hz (^1^H^N^) 143.8 ms | 16 | 0.150 | 2048 | 5100 | 1 d 17 h | 18.0 |
| 3D BT-(H)N(CA)NNH | 4000 Hz (^15^N)  28.3 ms | 2200 Hz (^15^N)  28.4 ms | | 14300 Hz (^1^H^N^) 143.8 ms | 16 | 0.150 | 2048 | 2700 | 1 d 20 h | 38.2 |

**Table S5** *Experimental parameters used for the acquisition of the NMR experiments on ID5_F2 for sequence-specific assignment*

|  | Spectral widths and maximal evolution times | | | | No. of scans | Inter-scan delays (s) | No. of complex points (aq) | No. of hypercomplex points | Duration of the experiment | Relative data points density (%) |
| --- | --- | --- | --- | --- | --- | --- | --- | --- | --- | --- |
| 2D BEST-TROSY | 2100 Hz (^15^N) 243.8 ms | | 10800 Hz (^1^H^N^) 95.1 ms | | 4 | 0.200 | 1024 | 512 | 35 min | 100.0 |
| 3D BT-HNCO | 2400 Hz (^13^C')  45.5 ms | 2100 Hz (^15^N)  51.9 ms | | 10800 Hz (^1^H^N^) 95.1 ms | 2 | 0.200 | 1024 | 7000 | 6 h 50 min | 57.9 |
| 3D BT-HN(CA)CO | 2400 Hz (^13^C')  45.5 ms | 2100 Hz (^15^N)  51.9 ms | | 10800 Hz (^1^H^N^) 95.1 ms | 4 | 0.200 | 1024 | 7000 | 13 h 40 min | 57.9 |
| 3D BT-HNCACB | 14000 Hz (^13^C^α/β^) 15.0 ms | 2100 Hz (^15^N)  51.9 ms | | 10800 Hz (^1^H^N^) 95.1 ms | 4 | 0.200 | 1024 | 9000 | 17 h 40 min | 38.8 |
| 3D BT-HN(CO)CACB | 14000 Hz (^13^C^α/β^) 15.0 ms | 2100 Hz (^15^N)  51.9 ms | | 10800 Hz (^1^H^N^) 95.1 ms | 4 | 0.200 s | 1024 | 9000 | 18 h 50 min | 38.8 |
| 3D BT-(H)N(COCA)NH | 2300 Hz (^15^N)  28.7 ms | 2100 Hz (^15^N)  51.9 ms | | 10800 Hz (^1^H^N^) 95.1 ms | 16 | 0.200 | 1024 | 3500 | 1 d 4 h  30 min | 47.5 |
| 3D BT-(H)N(CA)NNH | 2300 Hz (^15^N)  33.0 ms | 2300 Hz (^15^N)  33.0 ms | | 10800 Hz (^1^H^N^) 95.1 ms | 16 | 0.200 | 1024 | 3500 | 1 d 6 h | 59.0 |

**Table S6** *Experimental parameters used for the acquisition of the NMR experiments on ID5_F3 for sequence-specific assignment*

|  | Spectral widths and maximal evolution times | | | | No. of scans | Inter-scan delays (s) | No. of complex points (aq) | No. of hypercomplex points | Duration of the experiment | Relative data points density (%) |
| --- | --- | --- | --- | --- | --- | --- | --- | --- | --- | --- |
| 2D BEST-TROSY | 2000 Hz (^15^N) 128.0 ms | | 10800 Hz (^1^H^N^) 95.1 ms | | 4 | 0.20 | 1024 | 256 | 15 min | 100.0 |
| 3D BT-HNCO | 2400 Hz (^13^C') 52.9 ms | 2300 Hz (^15^N)  55.2 ms | | 10800 Hz (^1^H^N^) 95.1 ms | 2 | 0.200 | 1024 | 6000 | 5 h 50 min | 36.6 |
| 3D BT-HN(CA)CO | 2400 Hz (^13^C') 52.9 ms | 2300 Hz (^15^N)  55.2 ms | | 10800 Hz (^1^H^N^) 95.1 ms | 4 | 0.200 | 1024 | 6000 | 11 h 40 min | 36.6 |
| 3D BT-HNCACB | 14000 Hz (^13^C^α/β^) 15.0 ms | 2300 Hz (^15^N)  55.2 ms | | 10800 Hz (^1^H^N^) 95.1 ms | 4 | 0.200 | 1024 | 8000 | 15 h 40 min | 29.6 |
| 3D BT-HN(CO)CACB | 14000 Hz (^13^C^α/β^) 15.0 ms | 2300 Hz (^15^N)  55.2 ms | | 10800 Hz (^1^H^N^) 95.1 ms | 4 | 0.200 | 1024 | 8000 | 16 h 45 min | 29.6 |
| 3D BT-(H)N(COCA)NH | 2300 Hz (^15^N)  28.7 ms | 2300 Hz (^15^N)  55.2 ms | | 10800 Hz (^1^H^N^) 95.1 ms | 8 | 0.200 | 1024 | 5000 | 20 h 25 min | 58.3 |
| 3D BT-(H)N(CA)NNH | 2300 Hz (^15^N)  33.0 ms | 2300 Hz (^15^N)  33.0 ms | | 10800 Hz (^1^H^N^) 95.1 ms | 16 | 0.200 | 1024 | 3000 | 1 d 1 h  40 min | 50.5 |

**Table S7** Experimental parameters used for the NMR titration between full-length ID5 and IQGAP1-F

|  | Spectral widths and maximal evolution times | | No. of scans | Inter-scan delays (s) | No. of complex points (aq) | Duration of the experiment |
| --- | --- | --- | --- | --- | --- | --- |
| 2D BEST-TROSY | 2500 Hz (^15^N) 102.4 ms | 10200 Hz (^1^H^N^) 403.0 ms | 32 | 0.100 | 4096 | 2 h 20 min |

**Table S8** *ID5 constructs used*

Amino acid sequences of ID5 constructs used in this study (in comparison to Suppl. Figure S1, additional sequences derived from expression tags are indicated in italics).


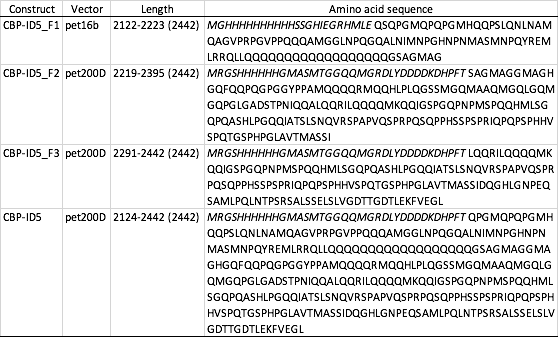


**References**

1. Liu, Z. et al. CPLM: a database of protein lysine modifications. *Nucleic Acids Res* **42,** D531-6 (2014)

2. Delorenzi, M. & Speed, T. An HMM model for coiled-coil domains and a comparison with PSSM-based predictions. *Bioinformatics* **18,** 617-25 (2002)

3. Dosztanyi, Z., Csizmok, V., Tompa, P. & Simon, I. IUPred: web server for the prediction of intrinsically unstructured regions of proteins based on estimated energy content. *Bioinformatics* **21,** 3433-4 (2005)

4. Cilia, E. et al. From protein sequence to dynamics and disorder with DynaMine. *Nat Commun* **4,** 2741 (2013)

5. Cilia, E. et al. The DynaMine webserver: predicting protein dynamics from sequence. *Nucleic Acids Res* **42,** W264-70 (2014)

6. Malhis, N., Jacobson, M. & Gsponer, J. MoRFchibi SYSTEM: software tools for the identification of MoRFs in protein sequences. *Nucleic Acids Res* **44,** W488-93 (2016)

7. Disfani, F. M. et al. MoRFpred, a computational tool for sequence-based prediction and characterization of short disorder-to-order transitioning binding regions in proteins. *Bioinformatics* **28,** i75-83 (2012)
